# Pro‐ATO/Allicin Liposomes for Dual‐Pathway Targeting of p53‐Mutant Tumors

**DOI:** 10.1002/advs.202519194

**Published:** 2026-01-29

**Authors:** Xiaoling Xu, WeiYi Cheng, Menghang Yang, Li He, WeiYe Ren, JingQuan Chen, LiTing He, Dandan Bao, Zhihong Zhu, Lixin Wang, Qinghua Yao, Ji‐Gang Piao

**Affiliations:** ^1^ Department of Radiation Oncology Shanghai Pulmonary Hospital Tongji University School of Medicine Shanghai China; ^2^ Zhejiang Provincial Key Laboratory of Diagnosis and Treatment of Thoracic Cancer Hangzhou Zhejiang China; ^3^ School of Pharmaceutical Sciences The First Affiliated Hospital of Zhejiang Chinese Medical University Hangzhou Zhejiang China; ^4^ Department of Dermatology & Cosmetology The First Affiliated Hospital of Zhejiang Chinese Medical University Hangzhou Zhejiang China; ^5^ Department of Integrative Medicine Shanghai Pulmonary Hospital Tongji University School of Medicine Shanghai China; ^6^ Department of Oncology The Second Affiliated Hospital of Zhejiang Chinese Medical University Xinhua Hospital of Zhejiang Province Hangzhou Zhejiang China; ^7^ Key Laboratory for Research on the Pathogenesis of ‘Inflammation‐Cancer Transformation’ in Intestinal Diseases Hangzhou Zhejiang China

**Keywords:** allicin, DNA damage response (DDR), Lung cancer, p53 mutation, Synthetic lethality

## Abstract

Mutations in the tumor suppressor p53 disrupt DNA damage response (DDR) and drive therapeutic resistance in lung cancer. Although arsenic trioxide (ATO) can restore transcriptional activity of structural p53 mutants, its clinical application is limited by subtype selectivity and systemic toxicity. In parallel, p53 deficiency creates dependence on S/G2 checkpoints, rendering ATR a synthetic lethal target; however, allicin, a natural ATR inhibitor and hydrogen sulfide (H_2_S) donor, suffers from poor stability and bioavailability. Here, we developed a liposomal nanomedicine co‐delivering pro‐ATO (As^5+^) and allicin (AsAcP@LP) to integrate mutant p53 reactivation with DDR‐targeted synthetic lethality. This formulation improves drug stability, pharmacokinetics, and tumor accumulation while masking allicin's odor. Upon tumor‐specific release, allicin‐mediated redox activation converts As^5+^ to cytotoxic As^3+^, enabling selective p53 reactivation, concurrent ATR inhibition, and H_2_S‐amplified apoptosis. AsAcP@LP exhibits synergistic antitumor efficacy with favorable tolerability, providing a rational nanotherapeutic strategy for p53‐mutant cancers.

## Graphical abstract

Schematic illustration of the “pro‐ATO”/allicin liposomal strategy. Liposomal encapsulation improves the stability and bioavailability of both agents while masking allicin's odor. Upon release in the tumor microenvironment, ATO reactivates structural p53 mutants, and allicin inhibits ATR signaling while releasing H_2_S, collectively inducing synthetic lethality. This dual‐action mechanism synergistically integrates p53 reactivation with DNA damage response inhibition, resulting in enhanced antitumor efficacy against p53‐mutant cancers.

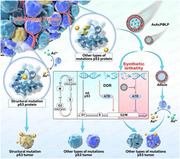

## 1 Introduction

The tumor suppressor p53 is a master transcription factor regulating cell‐cycle arrest, apoptosis, senescence, and DNA repair, thereby safeguarding genomic integrity [1, 2, 3]. Under physiological conditions, p53 protein levels are tightly controlled through MDM2‐mediated degradation, while cellular stress stabilizes and activates p53 to initiate tumor‐suppressive programs [4, 5, 6, 7, 8]. However, TP53 is mutated in over 50% of human cancers, resulting in loss of tumor‐suppressive function, defective DNA damage response (DDR), genomic instability, and poor clinical outcomes [9, 10, 11, 12, 13].

Given its central role in genome maintenance, restoring mutant p53 activity represents an attractive therapeutic strategy. Nonetheless, most p53 mutants lack druggable pockets and have long been considered undruggable [14, 15, 16, 17, 18, 19]. Recent studies have identified arsenic trioxide (ATO) as a rare small molecule capable of reactivating structural p53 mutants by coordinating with cysteine residues in the DNA‐binding domain and stabilizing the β‐sandwich fold, thereby restoring transcriptional activity [18, 19, 20, 21]. Despite this breakthrough, ATO exhibits subtype selectivity and dose‐limiting toxicity, restricting its broader clinical application.

An alternative and complementary strategy for targeting p53‐mutant tumors is synthetic lethality. Loss of p53 abolishes the G1/S checkpoint, forcing cancer cells to rely on S and G2/M checkpoints for survival under genotoxic stress. Consequently, inhibition of DDR kinases such as ATR can selectively eliminate p53‐deficient cells [22, 23, 24, 25, 26, 27, 28]. Among these targets, ATR has emerged as one of the most promising synthetic lethal vulnerabilities. Allicin, a bioactive garlic‐derived compound, has been reported to inhibit ATR signaling and induce apoptosis in multiple cancer models. In addition, allicin acts as an endogenous hydrogen sulfide (H_2_S) donor, further amplifying apoptotic signaling [29, 30, 31]. However, its instability, short half‐life, limited bioavailability, and strong odor severely constrain clinical translation.

To overcome these limitations, we developed a liposomal nanomedicine co‐delivering a prodrug form of ATO (As^5^^+^) and allicin (AsAcP@LP). Liposomal encapsulation enhances drug stability, improves pharmacokinetics, and enables tumor‐selective accumulation. Importantly, the formulation preserves arsenic in its less toxic pentavalent state during circulation, while enabling tumor microenvironment–triggered redox activation. Upon release, allicin inhibits ATR and generates a reductive milieu that converts As^5^^+^ to cytotoxic As^3^^+^, thereby coupling mutant p53 reactivation with DDR‐targeted synthetic lethality. This dual‐pathway strategy offers a rational and potentially translatable therapeutic approach for p53‐mutant cancers.

## 2 Results

### 2.1 Preparation and Characterization of AsAcP@LP

**FIGURE 1:**
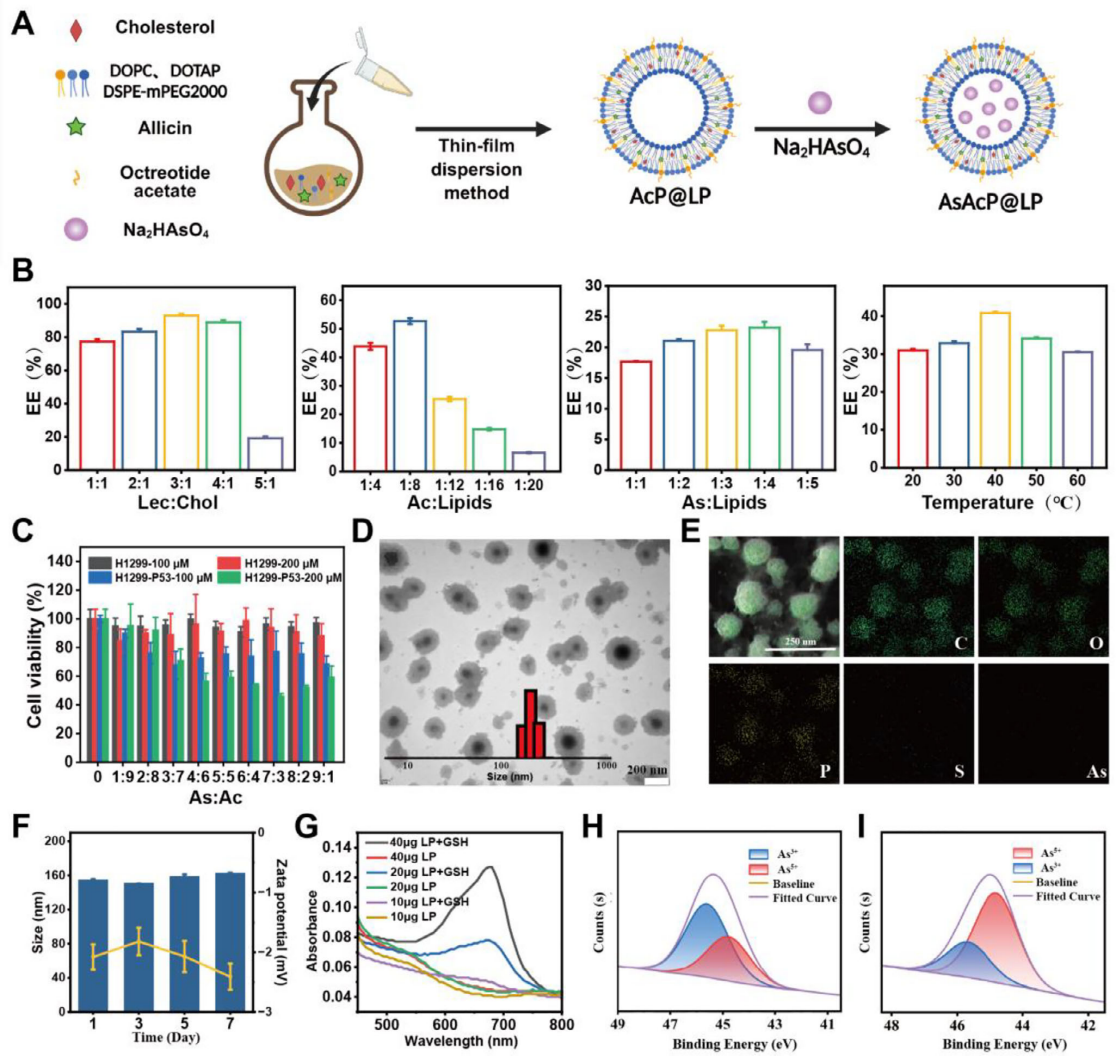
Preparation and characterization of AsAcP@LP. (A) Schematic overview of AsAcP@LP liposomal preparation via thin‐film hydration, optimizing parameters for drug loading and stability. (B) Influence of formulation parameters—phospholipid‐to‐cholesterol ratios, arsenic‐to‐lipid ratios, allicin‐to‐lipid ratios, and reaction temperature—on particle size, PDI, and encapsulation efficiency (*n* = 3). (C) Cytotoxicity evaluation of various molar ratios of As^5+^ to allicin in H1299‐P53 cells. (D) TEM image of AsAcP@LP. (E) EDS mapping showing elemental distribution of C, P, O, As, and S in AsAcP@LP. (F) Stability testing of AsAcP@LP stored at room temperature for 7 days, with monitoring of particle size, PDI, and zeta potential (*n* = 3). (G) H_2_S release profiles of AsAcP@LP in PBS with or without 10  mM GSH. (H) XPS spectra of As valence states in untreated AsAcP@LP. (I) XPS spectra of AsAcP@LP after GSH treatment.

Liposomes were selected as drug carriers due to their biocompatibility, nanoscale size favorable for passive tumor targeting via the EPR effect [32], and their ability to co‐encapsulate hydrophilic and hydrophobic molecules. Based on this rationale, we designed AsAcP@LP to co‐deliver pentavalent arsenic (As^5+^) and allicin, with the aim of enhancing their synergistic antitumor activity while improving drug stability and tumor accumulation. In this study, AsAcP@LP were prepared using the thin‐film hydration method (Figure 1A). To obtain a uniform particle size distribution, high drug encapsulation efficiency, and good formulation stability, key parameters, including phospholipid‐to‐cholesterol ratio, drug‐to‐lipid ratio, and reaction temperature, were optimized. Liposomes prepared with a phospholipid‐to‐cholesterol ratio of 3:1 showed the best size uniformity and the highest encapsulation efficiency (Figure 1B; Table S1). Further optimization identified an arsenic‐to‐lipid molar ratio of 1:4 and an allicin‐to‐lipid mass ratio of 1:8 as optimal, yielding liposomes with high encapsulation efficiency and good dispersity (Figure 1B; Tables S2 and S3). Reaction temperature was also critical, with 40°C producing the most stable formulation and the most favorable particle size distribution (Figure 1B; Table S4). Overall, these optimized parameters produced liposomes with high stability, efficient drug loading, and desirable particle characteristics.

To further validate the rationality of the drug ratio between pentavalent arsenic (As^5+^) and allicin within the liposomal formulation and assess their synergistic antitumor potential, we first evaluated the cytotoxic selectivity of various drug ratios in p53‐expressing H1299 cells (H1299‐P53). Among the tested combinations, a 7:3 molar ratio at a total concentration of 200 µM produced the strongest growth‐inhibitory effect and the lowest cell viability (Figure 1C). This finding indicates a pronounced synergistic cytotoxic effect and, importantly, is consistent with the ratio encapsulated in the liposomal formulation, thereby supporting the rationale of our formulation design.

To comprehensively characterize the physicochemical properties of AsAcP@LP, transmission electron microscopy (TEM) revealed that the liposomes had a near‐spherical morphology with well‐defined boundaries and uniform particle sizes, displaying a typical bilayer vesicular structure (Figure 1D). Particle sizes were predominantly distributed between 100–200 nm, which is suitable for passive tumor targeting via the enhanced permeability and retention (EPR) effect. These observations confirm the feasibility and effectiveness of the thin‐film hydration for producing nanosized liposomes. To further assess drug distribution within the liposomes, elemental mapping by energy‐dispersive spectroscopy (EDS) was performed in conjunction with TEM. High signals for C, P, and O were detected, consistent with the primary components of the liposomal membrane. In addition, As and S were homogeneously distributed throughout the liposomes without noticeable aggregation or phase separation, suggesting a rational drug loading strategy and stable incorporation of active compounds (Figure 1E). Dynamic light scattering measurements confirmed narrow size distribution (PDI < 0.3) and good uniformity, while storage stability tests demonstrated minimal changes in particle size, zeta potential, and PDI over 7 days (Figure 1F). In summary, the AsAcP@LP exhibited well‐maintained structural integrity, uniform drug distribution, and favorable storage stability, providing a solid foundation for their application in cancer therapy. To further contextualize these properties, it is necessary to clarify the chemical form, encapsulation strategy, and stability of arsenic within the AsAcP@LP formulation, as well as their implications for controlled release and biological activity.This rational compartmentalization endows the AsAcP@LP system with a high degree of chemical stability during storage and systemic circulation, effectively maintaining arsenic predominantly in its less reactive pentavalent state (As^5+^) and thereby minimizing premature activation and off‐target toxicity. Upon cellular internalization, however, the elevated glutathione (GSH) levels characteristic of the tumor microenvironment disrupt liposomal integrity and trigger the release of allicin, which acts as an H_2_S donor and redox modulator. The locally generated reductive milieu facilitates the conversion of As^5+^ to the more cytotoxic trivalent form (As^3+^), enabling selective activation of the arsenic prodrug within tumor cells. This GSH‐responsive transformation not only ensures spatially confined drug activation but also functionally couples redox imbalance with arsenic‐mediated p53 reactivation and DNA damage amplification. Collectively, this cascade—from formulation‐stabilized As^5+^ preservation, to tumor‐specific reductive triggering, and ultimately to As^3+^‐driven cytotoxic signaling—establishes a closed mechanistic loop that underpins the controlled release behavior and enhanced antitumor efficacy of the AsAcP@LP platform.

To validate the tumor‐specific, microenvironment‐responsive release, we further investigated the release profile and arsenic valence‐state transformation of AsAcP@LP under GSH‐rich conditions. Because intracellular GSH concentrations are substantially higher in tumor cells than in normal tissues, we simulated the reductive tumor microenvironment by incubating liposomes in PBS with or without 10 mM GSH. We monitored H_2_S release and the reduction of pentavalent arsenic (As^5^^+^) within the system. The liposomes remained structurally stable under neutral conditions with negligible H_2_S release, indicating good integrity and encapsulation. In contrast, H_2_S release increased markedly under reductive conditions, suggesting liposomal disruption and confirming the system's responsiveness to tumor‐relevant stimuli (Figure 1G). To assess the GSH‐triggered reduction of As^5^^+^ to trivalent arsenic (As^3^^+^), X‐ray photoelectron spectroscopy (XPS) was performed to determine arsenic valence states before and after incubation. High‐resolution As 3d spectra indicated that the untreated liposomes contained approximately 63% As^5^^+^ and 37% As^3^^+^ (Figure 1H). This partial presence of As^3^^+^ in the initial formulation may stem from minor reduction events during liposome preparation or storage. Notably, upon exposure to GSH, the proportion of trivalent arsenic (As^3^^+^) markedly increased to 72%. This indicates that GSH effectively mediates liposome degradation and triggers the release of allicin as an H_2_S donor, thereby facilitating the reduction of pentavalent arsenic (As^5^^+^) to trivalent arsenic (As^3^^+^) (Figure 1I). These findings confirm that the encapsulated As^5^^+^ prodrug can be selectively activated in reductive tumor microenvironments, enhancing cytotoxicity and therapeutic potential. In summary, AsAcP@LP exhibits favorable structural stability, efficient drug encapsulation, GSH‐triggered release, and redox‐activated conversion, offering a promising strategy for tumor‐selective, high‐efficacy arsenic‐based cancer therapy and aligning with the growing demand for smart, microenvironment‐responsive drug delivery systems.

### 2.2 Cellular Pharmacodynamics of AsAcP@LP

**FIGURE 2:**
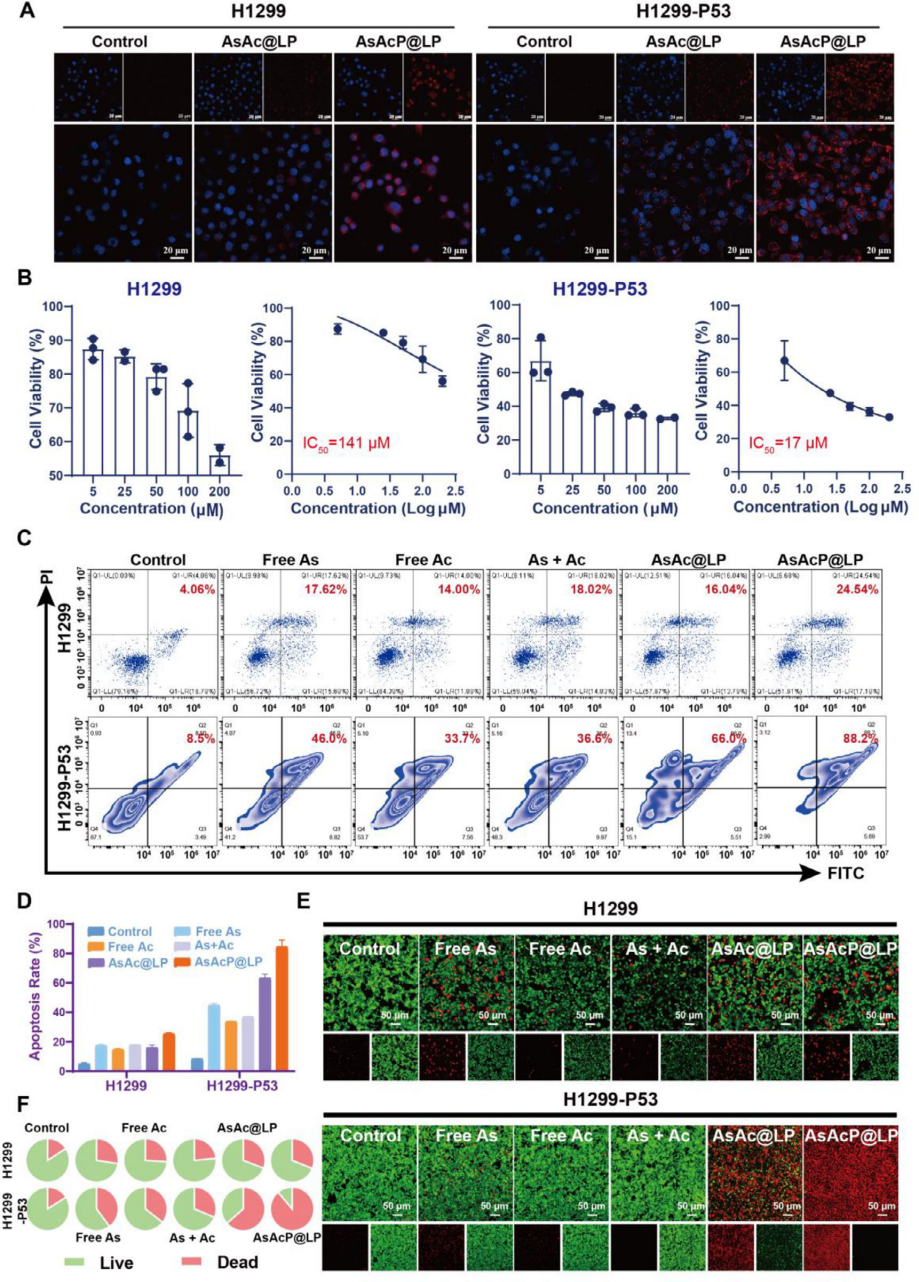
Cellular pharmacodynamics of AsAcP@LP. (A) CLSM images of Cy5.5‐labeled liposome uptake in H1299 and H1299‐P53 cells after 4 h. Scale bar = 20 µm. (B) Cytotoxicity of AsAcP@LP in varying concentrations for 24 h in H1299 and H1299‐P53 cells (*n* = 3). (C,D) Flow cytometry analysis of apoptosis in H1299 cells post 24‐h treatment with control, free As, free Ac, free As + Ac combination, AsAc@LP, and AsAcP@LP (*n* = 3). (E‐F) Fluorescence microscopy of live (green) and dead (red) H1299 and H1299‐P53 cells after 24‐h treatment, with pie charts showing cell viability across formulations. Scale bar = 50 µm.

To evaluate the cytotoxicity and biological performance of the lipid‐based delivery system, we first examined the cellular uptake of AsAcP@LP to confirm tumor cell internalization efficiency. A near‐infrared fluorescent probe (Cy5.5) was conjugated to the liposomes to enable real‐time visualization. As shown by CLSM images (Figure 2A), after 4 h of incubation, AsAcP@LP‐Cy5.5 exhibited significantly stronger red fluorescence in the cytoplasm of both cell lines than AsAc@LP–Cy5.5, indicating enhanced cellular uptake and efficient intracellular delivery. Notably, within each formulation group, the fluorescence distribution patterns were comparable between H1299 and H1299‐P53 cells, suggesting similar baseline internalization in the two models. This provides an important basis for subsequent cytotoxicity comparisons by minimizing potential confounding effects arising from differential uptake. Moreover, the enhanced intracellular accumulation of AsAcP@LP‐Cy5.5 implies that the improved performance may be attributed to surface ligand modifications, which may promote membrane binding and endocytosis and enhance tumor cell‐specific targeting [33, 34, 35]. Together, these results support the promise of this delivery system for efficient and selective therapeutic action.

To further evaluate the antitumor potential of AsAcP@LP, we performed a dose‐dependent cytotoxicity assay in H1299 and H1299‐P53 cells. As shown in Figure 2B, AsAcP@LP exerted a markedly stronger inhibitory effect in H1299‐P53 cells than in H1299 cells, indicating a pronounced p53‐dependent antitumor activity. At a concentration of 17 µM, the viability of H1299‐P53 cells decreased to approximately 50%, corresponding to an nearly eightfold greater suppression compared with that observed in H1299 cells. Importantly, this enhanced cytotoxicity toward p53‐expressing tumor cells did not translate into increased damage to normal cells. AsAcP@LP exhibited minimal cytotoxicity toward noncancerous Beas‐2B and HUVEC cells, with cell viabilities remaining above 80% even at concentrations up to 100 µm, suggesting good biocompatibility (Figure S1). Together, these results demonstrate that AsAcP@LP achieves selective antitumor efficacy while sparing normal cells, thereby highlighting a favorable therapeutic window.

To investigate the cellular mechanism underlying the antitumor activity of AsAcP@LP, apoptosis was quantified by Annexin V–FITC/PI dual‐staining flow cytometry in H1299 and H1299‐P53 cells. In H1299 cell, the percentage of late apoptotic cells in each group was as follows: Control, 4.06%; Free As, 17.62%; Free Ac, 14.00%; As + Ac, 16.04%; AsAc@LP, 18.02%; and AsAcP@LP, 24.54%. In contrast, the H1299‐P53 model exhibited the following late apoptosis rates: Control, 8.5%; Free As, 46.0%; Free Ac, 33.7%; As + Ac, 36.6%; AsAc@LP, 66.0%; and AsAcP@LP, 88.2% (Figure 2C,D). These results indicate that AsAcP@LP induces apoptosis more effectively than monotherapies and unmodified liposomes in both cell models, consistent with a strong synergistic effect. Notably, the pro‐apoptotic efficacy was markedly amplified in the H1299‐P53 cells, indicating enhanced therapeutic responsiveness in p53‐enriched molecular contexts. Overall, the superior apoptosis‐inducing capacity of AsAcP@LP provides mechanistic support for its increased cytotoxicity and supports further mechanistic studies and in vivo therapeutic evaluation.

To further verify the antitumor efficacy of AsAcP@LP at the cellular level, we employed a fluorescence‐based live/dead cell staining assay to visualize the viability status of treated cells. Following the indicated treatments, fluorescence imaging revealed the strongest red signal in AsAcP@LP‐treated H1299‐P53 cells, indicating pronounced cytotoxicity and effective induction of cell death. Although red fluorescence was also increased in H1299 cells treated with AsAcP@LP, the overall intensity was substantially lower (Figure 2E,F). This suggests a limited capacity of AsAcP@LP to induce cell death in the p53‐deficient context, implying that its antitumor activity may be closely related to specific intracellular signaling states, particularly p53 expression. Notably, these observations are consistent with the apoptosis data presented earlier, both highlighting the enhanced cell death‐inducing potential of AsAcP@LP in p53‐active tumor cells. Together, these findings reinforce the proposed synergistic mechanism of action of AsAcP@LP and provide indirect evidence supporting its ability to induce synthetic lethality in tumors with defined molecular characteristics.

### 2.3 Intracellular Mechanism of Action of AsAcP@LP

**FIGURE 3:**
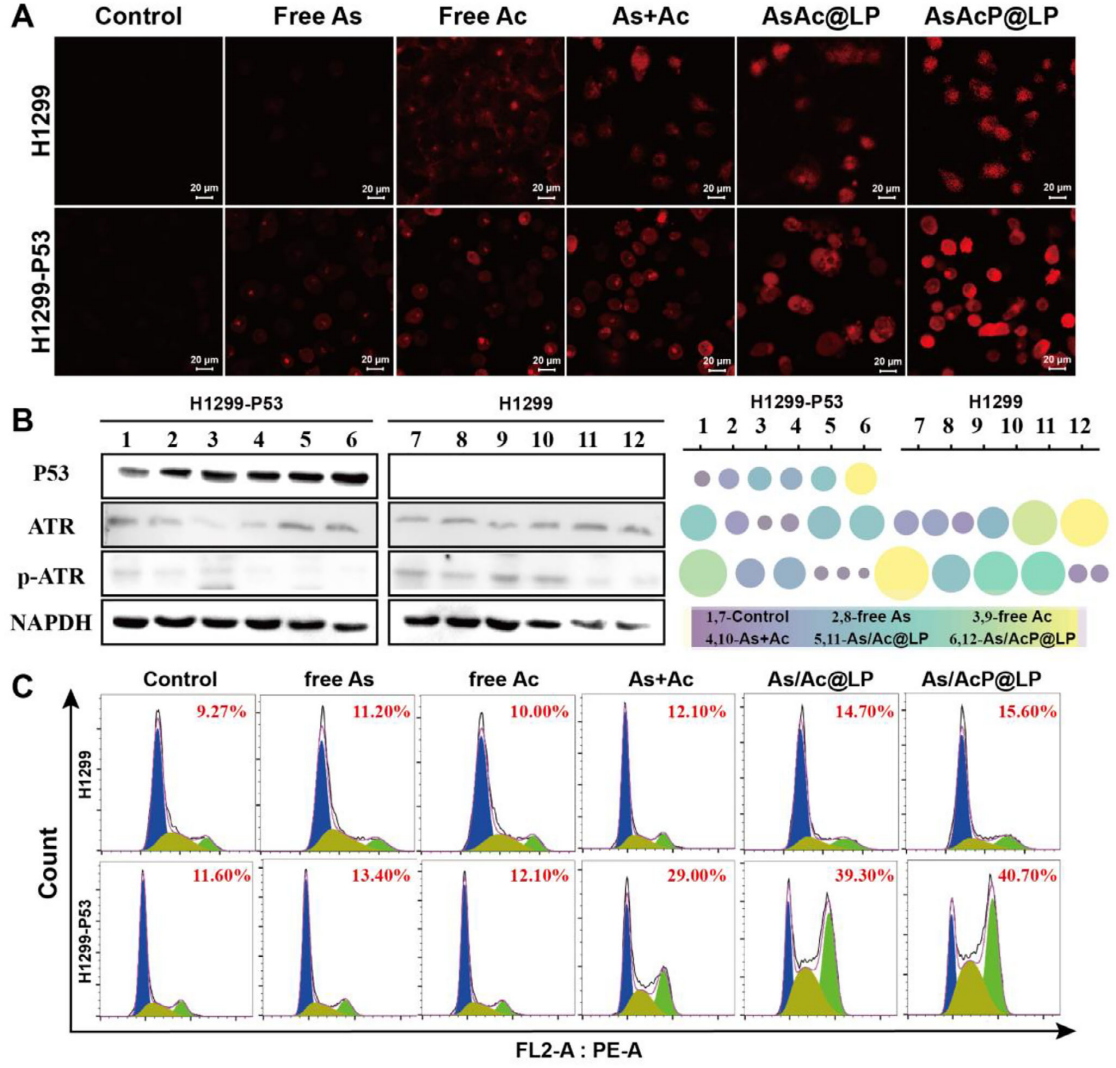
Investigation of intracellular synthetic lethality and drug efficacy. (A) Confocal images of H_2_S production in H1299‐P53 cells after treatment with Control, Free As, Free Ac, As + Ac, AsAc@LP, and AsAcP@LP using a red fluorescent H_2_S probe. Nuclei are DAPI‐stained (blue). Scale bar = 20 µm. (B) Western blot analysis of p53, ATR, and phosphorylated ATR (p‐ATR) in H1299‐P53 cells after different treatments. (C) Cell cycle distribution (G_2_/M phase) in H1299 and H1299‐P53 cells post 24‐h treatment, analyzed by flow cytometry with PI staining.

To investigate the intracellular response mechanism of AsAcP@LP, the levels of H_2_S generation across different treatment groups were visually compared. H_2_S, a typical gaseous signaling molecule, plays essential roles in apoptosis, redox regulation, and remodeling of the tumor microenvironment. Therefore, its intracellular release level can serve as an indirect indicator of the activation efficiency of allicin‐derived compounds. Both the Control and Free As groups exhibited weak red fluorescence in H1299‐P53 cells, indicating low levels of endogenous H_2_S production in the absence of allicin or ineffective intracellular delivery (Figure 3A). Moderate red fluorescence was observed in the Free Ac and As + Ac groups without significant difference, suggesting that free allicin alone exhibits limited intracellular activation, and its combination with arsenic does not notably enhance H_2_S release. In contrast, the AsAc@LP group displayed markedly enhanced red fluorescence, indicating that liposomal encapsulation facilitates improved intracellular accumulation and partial activation of allicin. Notably, the AsAcP@LP group exhibited the strongest red fluorescence, significantly higher than the other groups, suggesting that this formulation enables more efficient intracellular delivery and GSH‐responsive H_2_S release. These findings are consistent with the previous cellular uptake results, confirming that AsAcP@LP not only enhances targeted endocytosis of tumor cells but also promotes the efficient activation and release of allicin under the highly reductive tumor microenvironment, thereby significantly increasing intracellular H_2_S levels.

To further elucidate the molecular mechanism underlying the antitumor effect of AsAcP@LP, particularly whether it exerts a “synthetic lethality” effect by interfering with DNA damage repair pathways, we further examine the expression of key signaling proteins, including p53, ATR, and phosphorylated ATR (p‐ATR), across different treatment groups (Figure 3B). The results showed increased expression of p53 in protein levels in H1299‐P53 cells following AsAcP@LP treatment. To assess whether the elevated levels of p53 protein correspond to functional reactivation, we quantified the expression of canonical downstream targets of p53 using ELISA. As illustrated in Figure S2, treatment with AsAcP@LP led to a significant upregulation of p21 and Bax protein levels in H1299‐P53 cells compared to untreated controls. Consistent with these findings, Western blot (WB) analysis also showed a similar trend. These results demonstrate that p53 is transcriptionally active and capable of initiating downstream signaling for cell cycle arrest and apoptosis. This suggests that AsAcP@LP might restore the function of mutant p53, reducing its abnormal accumulation and activity in tumor cells, and thereby enhancing cellular sensitivity to DNA damage. In contrast, total ATR expression remained unchanged across all groups, indicating that liposomal treatment did not significantly affect overall ATR levels. In the AsAcP@LP treatment group, the phosphorylated ATR (p‐ATR) was reduced, indicating a suppression of the ATR signaling pathway under this condition. As a central kinase in the DNA damage response, ATR is typically activated in response to replication stress or DNA double‐strand breaks. Thus, the observed decrease in p‐ATR suggests that liposome‐induced stress may trigger but ultimately impair the cellular DNA damage response. Taken together, AsAcP@LP appears to induce a “synthetic lethality” effect by simultaneously restoring mutant p53 function and inhibiting ATR signaling, thereby selectively eliminating tumor cells. This mechanism not only underscores the precision therapeutic potential of AsAcP@LP but also provides a strong theoretical rationale for its further application in p53‐deficient cancers.

To further elucidate the antitumor effects of AsAcP@LP, we systematically assessed its effects on cell‐cycle distribution. Cell‐cycle disruption is a major therapeutic mechanism of many anticancer agents, and G_2_/M phase arrest is commonly associated with DNA damage response, reflecting the cellular checkpoint control before mitotic entry to ensure genomic integrity. As shown in Figure 3C, the proportion of cells in the G_2_/M phase varied significantly across different treatment groups in both H1299 and H1299‐P53 cell lines. In H1299 cells, the G_2_/M population percentages for the Control, Free As, Free Ac, As + Ac, AsAc@LP, and AsAcP@LP groups were 9.27%, 11.20%, 10.00%, 12.10%, 14.70%, and 15.60%, respectively. In contrast, the corresponding values in H1299‐P53 cells were 11.60%, 13.40%, 12.10%, 29.00%, 39.30%, and 40.70%, respectively. These results demonstrate that AsAcP@LP treatment significantly induces G_2_/M phase arrest, particularly in H1299‐P53 cells, in which the G_2_/M fraction reached 40.70%, exceeding that observed with the other treatments. Even in p53‐null H1299 cells, AsAcP@LP induced a moderate increase in G_2_/M accumulation, suggesting a robust capacity for cell cycle interference. This effect is likely related to the induction of DNA damage and activation of the ATR signaling pathway. On one hand, As^5+^ may cause oxidative stress and replication instability, contributing to DNA damage accumulation. On the other hand, allicin released intracellularly may enhance the stress response, synergistically act on cell cycle checkpoints and promoting ATR pathway activation. This cooperative interaction leads to cell cycle arrest at the G_2_/M phase, preventing damaged DNA from progressing into mitosis. Moreover, the arrest effect was more pronounced in the H1299‐P53 model, indicating that p53 expression enhances cellular sensitivity to AsAcP@LP‐induced stress and reinforces the synthetic lethality between p53 and ATR pathways. These findings provide cell cycle‐level evidence for the mechanism of AsAcP@LP and highlight its potential as a synthetic lethality‐based drug delivery system for targeted cancer therapy.

**FIGURE 4:**
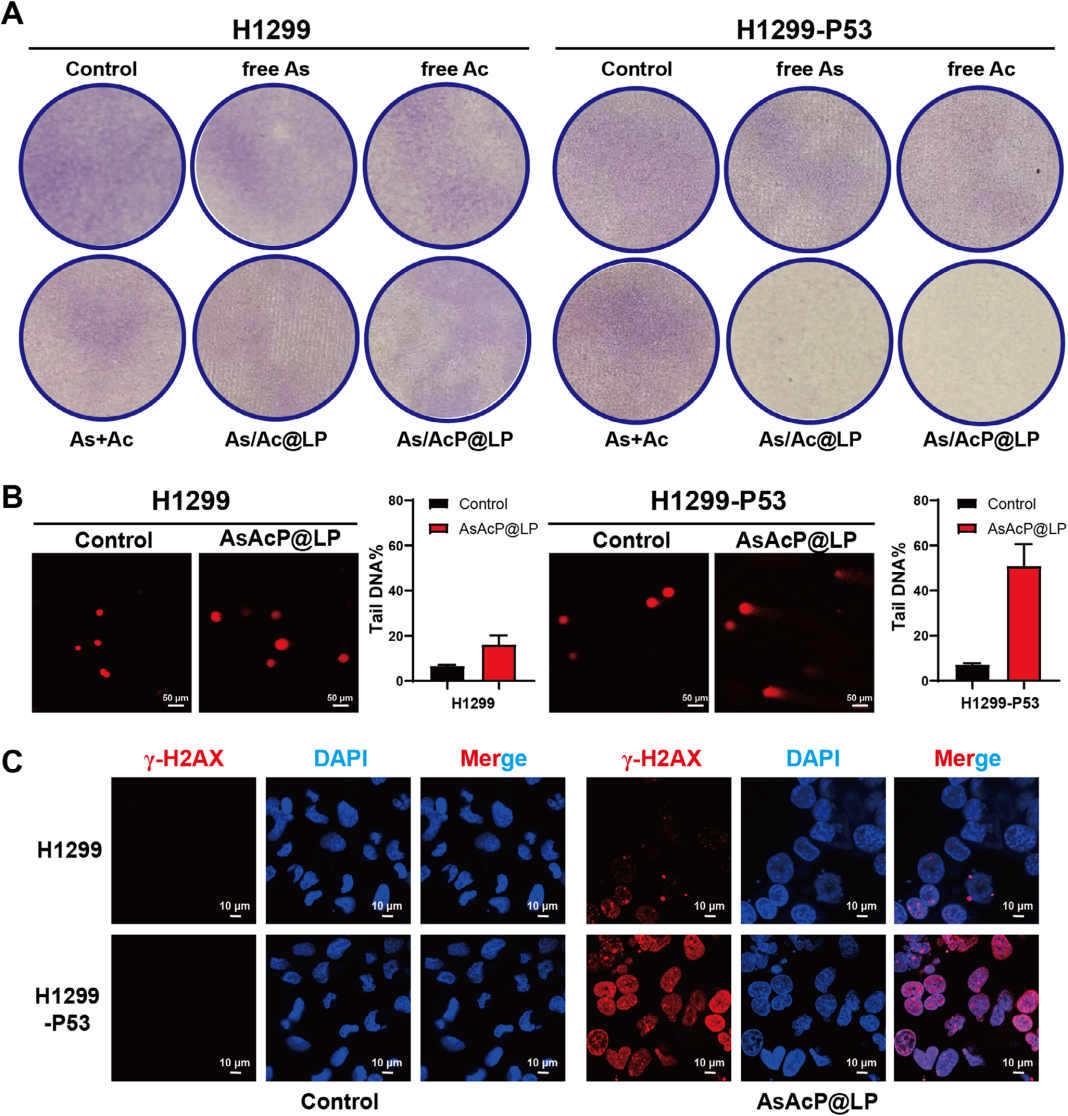
Investigation of the cellular effects induced by DNA damage. (A) Representative images of colony formation in H1299 and H1299‐P53 cells following treatment with different formulations. (B) Visualization of DNA damage levels in H1299 and H1299‐P53 cells as shown by comet assay images. Tail DNA percentage was quantified using CASP software. Scale bars = 50 µm. (C) Immunofluorescence staining of γ‐H2AX (green) and nuclei (DAPI, blue) to detect DNA double‐strand breaks. Scale bars = 10 µm.

To comprehensively evaluate the DNA damage induced by AsAcP@LP in H1299 and H1299‐P53 cells, a series of complementary analyses were performed. As shown in Figure 4A, AsAcP@LP markedly reduced the proliferative capacity of H1299‐P53 cells, which consistently exhibited the lowest viability across all treatment groups, indicating strong growth‐inhibitory effects on p53‐overexpressing cells. Comet assay images showed minimal DNA abnormalities in control groups, consistent with a low baseline level of damage (Figure 4B). In contrast, AsAcP@LP‐treated H1299‐P53 cells displayed pronounced DNA fragmentation, evidenced by elongated comet tails. Quantitatively, % tail DNA remained below 10% in both control and treated H1299 cells, indicating only mild damage, whereas H1299‐P53 cells treated with AsAcP@LP exhibited nearly 50% tail DNA, consistent with severe DNA disruption. In line with these findings, γ‐H2AX—a sensitive marker of DNA double‐strand breaks—was barely detectable in untreated cells but was robustly upregulated in AsAcP@LP‐treated H1299‐P53 cells, with increased fluorescence intensity and a higher number of foci (Figure 4C). Collectively, these findings demonstrate that the AsAcP@LP formulation induces significant DNA damage, particularly in cells harboring mutant p53. The enhanced cytotoxicity is likely attributed to a synthetic lethality effect arising from the interplay between p53 mutation and ATR pathway activation.

### 2.4 Transcriptome Sequencing Analysis

**FIGURE 5:**
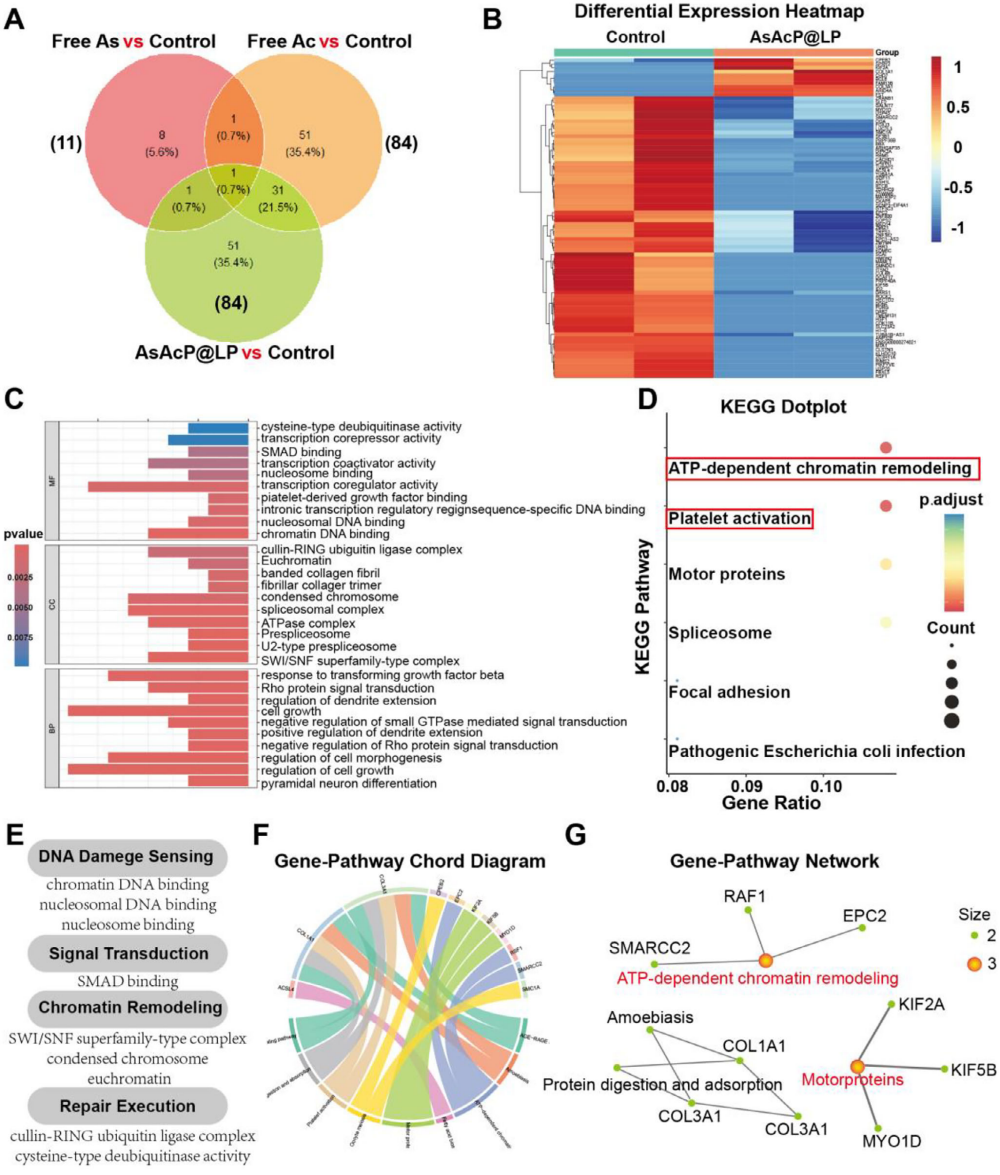
Transcriptome Sequencing Analysis. (A) Venn diagram showing the overlap of differentially expressed genes (DEGs) among AsAcP@LP vs Control, Free As vs Control, and Free Ac vs Control groups. (B) Heatmap of DEGs in AsAcP@LP vs Control, highlighting transcriptional reprogramming and distinct expression patterns. (C, E) Gene Ontology (GO) enrichment analysis of DEGs demonstrates significant involvement in DDR‐related processes. (D) KEGG pathway analysis indicates clustering of DEGs in ATP‐dependent chromatin remodeling and spliceosome pathways. (F, G) Chord diagrams mapping 51 unique DEGs in AsAcP@LP‐treated cells.

Encouraged by the pronounced therapeutic effect of AsAcP@LP, we further performed RNA‐seq analysis to elucidate its potential antitumor mechanism. AsAcP@LP revealed that the gene expression changes induced by AsAcP@LP vs Control shared only 2 overlapping differentially expressed genes (DEGs) with the Free As vs Control group and 32 with the Free Ac vs Control group, with FAM13B being the sole common DEG across all comparisons (Figure 5A).

Heatmap analysis (Figure 5B) revealed clear transcriptional distinctions between AsAcP@LP and Control, indicating robust treatment‐induced transcriptional reprogramming. Several genes linked to DDR and chromatin regulation were differentially expressed, including RSF1, SMARCC2, and SMC1A, key components of chromatin remodeling and cohesion complexes. Altered expression was also observed for USP43, CUL4B, and FBXL5, which regulate ubiquitination and deubiquitination pathways. In addition, spliceosome‐associated genes such as SF3B1, PRPF38B, and RBM5 were perturbed. Among the 84 DEGs identified in the AsAcP@LP versus Control comparison, 75 were downregulated and 9 were upregulated (Figure S3). Gene Ontology (GO) enrichment analysis (Figure 5C,E) revealed significant enrichment of DNA damage response–related terms, including “DNA/nucleosomal binding”, “SWI/SNF chromatin remodeling”, “Cullin‐RING ligase/deubiquitinase activity”, “SMAD binding”, and “spliceosome” covering processes from lesion recognition and chromatin remodeling to signal transduction and repair. KEGG pathway analysis (Figure 5D) further confirmed clustering of DEGs in ATP‐dependent chromatin remodeling and spliceosome pathways, with additional enrichment observed in focal adhesion and motor protein pathways. Collectively, these results demonstrated that AsAcP@LP enhanced genotoxic stress by both inducing DNA damage and impairing repair resolution.

To further dissect AsAcP@LP‐specific alterations, we analyzed 51 unique DEGs. Chord diagram mapping (Figure 5F,G) highlighted several DDR‐related modules. In particular, RSF1, SMARCC2, and SMC1A were linked to the ATP‐dependent chromatin remodeling pathway, emphasizing potential disruption of chromatin accessibility and structural maintenance during DDR. Motor protein genes (KIF2A, KIF5B, MYO1D) were associated with motor protein pathways, suggesting altered intracellular transport that may affect repair factor trafficking. Structural genes such as COL1A1, COL3A1, and EPC2 were enriched in platelet activation and extracellular matrix–related pathways, reflecting broader effects on adhesion and survival signaling. Taken together, these results provide strong mechanistic evidence that AsAcP@LP not only induces DNA lesions but also compromises their repair by interfering with chromatin remodeling and associated regulatory processes, thereby offering new insights into targeting DDR pathways in cancer therapy.

### 2.5 In Vivo Pharmacodynamics

**FIGURE 6:**
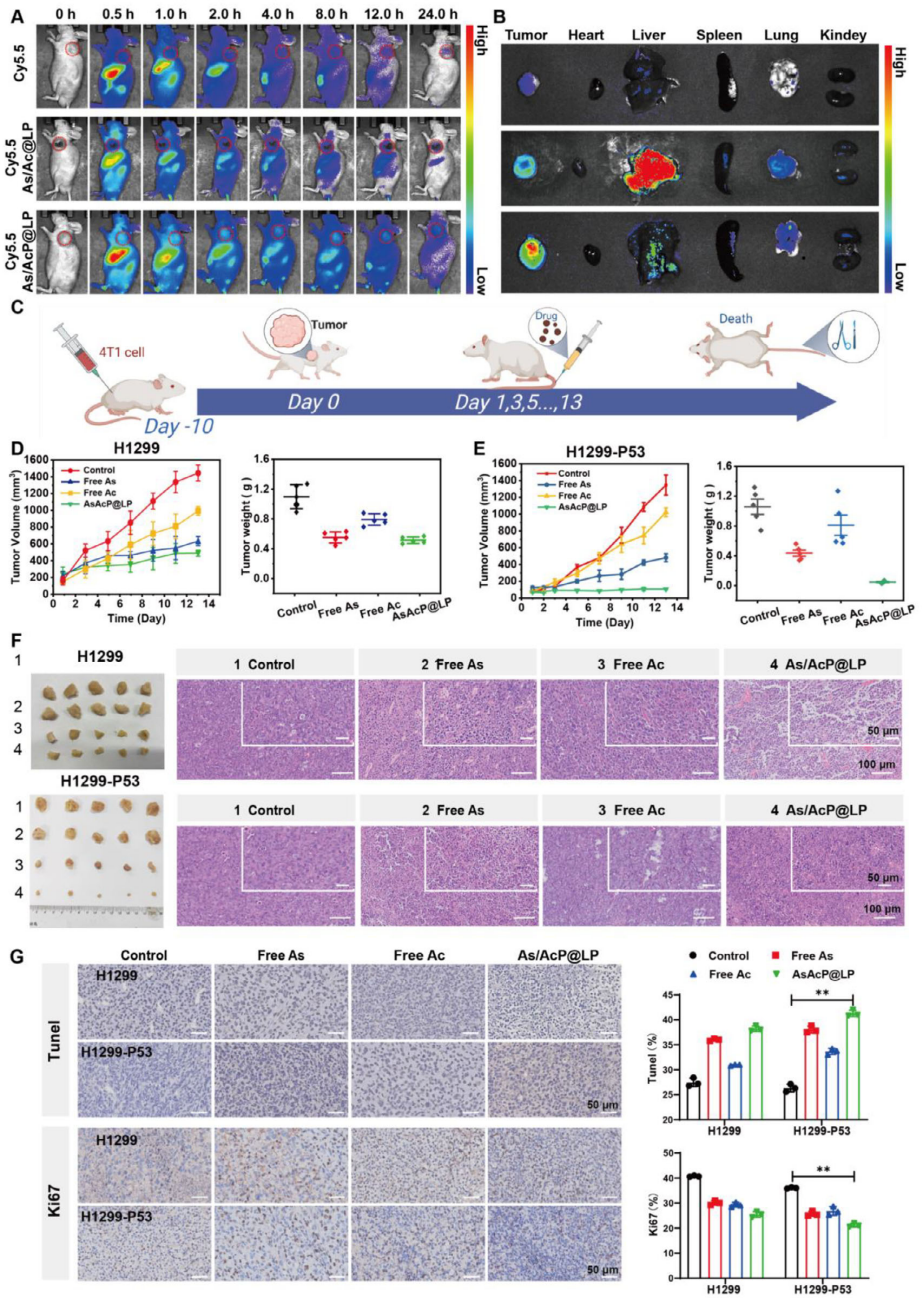
In vivo pharmacodynamics. (A) In vivo fluorescence imaging of Free Cy5.5, Cy5.5‐labeled AsAc@LP, and AsAcP@LP at different time points post‐intravenous injection. (B) Ex vivo fluorescence intensity of tumors and major organs. (C) Schematic of in vivo treatment regimen for tumor‐bearing mice. (D, E) Tumor growth monitoring during treatment and tumor weight measurement at study endpoint. (F) H&E staining of tumor sections. (G) TUNEL and Ki67 immunohistochemical staining of tumor sections.

To evaluate the in vivo tumor‐targeting capability of AsAcP@LP, we constructed a near‐infrared fluorescent liposomal probe by labeling AsAcP@LP with Cy5.5, and monitored its biodistribution in nude mice using a small‐animal in vivo imaging system. In the free Cy5.5 group, the fluorescence signal diminished rapidly, with only faint accumulation observed in excised tumors, indicating that the free dye underwent rapid systemic clearance and lacked either passive or active tumor‐targeting ability. In contrast, Cy5.5‐labeled AsAc@LP and AsAcP@LP gradually accumulated at the tumor site after intravenous injection, reaching peak fluorescence intensity at approximately 8 h post‐injection. Although the signal gradually decreased thereafter, significant fluorescence retention within the tumor was still detectable at 24 h, with AsAcP@LP exhibiting stronger tumor accumulation than AsAc@LP (Figure 6A,B). Ex vivo imaging further confirmed that the AsAcP@LP group displayed the highest level of tumor accumulation (Figure S4). These findings demonstrate that AsAcP@LP possesses superior in vivo stability and tumor‐targeting capability, enabling prolonged circulation in the bloodstream and efficient enrichment within tumor tissues via the enhanced permeability and retention (EPR) effect. Moreover, the incorporation of DSPE‐PEG2000 on the liposomal surface likely reduced clearance by the mononuclear phagocyte system (MPS), thereby extending circulation time and further improving passive tumor targeting efficiency. Collectively, these results provide a solid foundation for subsequent pharmacodynamic evaluation of liposomal formulations in vivo.

Building on the potent antitumor activity of AsAcP@LP observed in vitro, we further evaluated its therapeutic efficacy and biosafety in vivo using H1299 and H1299‐P53 tumor‐bearing nude mouse models. Tumor‐bearing mice were randomly divided into four groups and treated with saline, Free As, Free Ac, or AsAcP@LP. Mice received intravenous injections every other day for a total of seven doses, with body weight and tumor volume recorded every two days (Figure 6C). Body weight remained stable across all treatment groups throughout the dosing period (Figure S5), suggesting that none of the treatments induced significant systemic toxicity. Notably, the AsAcP@LP group maintained antitumor efficacy while exhibiting favorable biocompatibility. Tumor growth curves and final tumor weights further confirmed the superior therapeutic effect of AsAcP@LP in H1299‐P53 xenografts (Figure 6D,E). At the end of treatment, tumors in the PBS group exhibited rapid progression with an average weight of 1.058 g. Free Ac showed limited tumor suppression (0.810 g), while Free As demonstrated moderate antitumor activity (0.436 g). In contrast, AsAcP@LP achieved the most pronounced inhibition, with an average tumor weight of only 0.048 g, consistent with the markedly delayed tumor growth observed during treatment. Together, these results indicate that AsAcP@LP not only enhances the synergistic antitumor effect of pentavalent arsenic and allicin but also mitigates the rapid clearance and nonspecific toxicity associated with their free forms, thereby improving the overall therapeutic index. Collectively, AsAcP@LP exhibited outstanding in vivo antitumor efficacy and safety, supporting its potential as a cooperative nanocarrier system for cancer therapy.

To further visualize the antitumor efficacy of AsAcP@LP at the tissue level, tumor sections from each group were subjected to H&E, TUNEL, and Ki67 immunohistochemical staining to assess morphological changes, apoptosis, and proliferative activity, respectively. H&E staining revealed that tumors in the control group maintained intact architecture with densely packed, regularly arranged nuclei and negligible necrosis (Figure 6F). In contrast, drug‐treated groups displayed varying degrees of nuclear pyknosis, chromatin condensation, and cytoplasmic vacuolization, indicating significant pathological damage. Notably, AsAcP@LP treatment induced the most profound histological alterations, characterized by extensive intercellular space expansion, nuclear shrinkage, and widespread necrotic regions. TUNEL staining further demonstrated apoptotic induction, with DNA fragmentation visualized as brown‐positive nuclei (Figure 6G). Control tumors exhibited minimal TUNEL positivity, reflecting low basal apoptosis, whereas all treatment groups showed markedly increased apoptotic signals. Among them, AsAcP@LP treatment resulted in the highest number of TUNEL‐positive cells, suggesting strong activation of apoptosis pathways and effective induction of tumor cell death. Consistently, Ki67 staining revealed the highest proportion of proliferative cells in the control group, confirming the aggressive proliferative nature of untreated tumors. Drug‐treated tumors showed a pronounced reduction in Ki67‐positive nuclei, with AsAcP@LP treatment yielding the weakest proliferative signal, where only a small fraction of cells remained in active cycling. These findings highlight the superior ability of AsAcP@LP to induce apoptosis and suppress tumor proliferation at the pathological level.

### 2.6 Safety Evaluation

In addition to assessing the in vivo antitumor efficacy, we systematically evaluated the safety profile of AsAcP@LP through hematological analysis, serum biochemistry, and histopathological examination of major organs. Routine blood parameters showed no significant intergroup differences and remained within normal ranges, indicating the absence of hematological toxicity (Figures S6 and S7). Similarly, serum biochemical indices revealed no significant deviations from control levels, suggesting that AsAcP@LP did not impair hepatic or renal function. Moreover, histological analysis of the heart, liver, spleen, lungs, and kidneys by H&E staining revealed no apparent pathological alterations across treatment groups, further confirming the absence of overt organ toxicity (Figures S4 and S5). Collectively, these findings demonstrate that AsAcP@LP possesses excellent biocompatibility and favorable safety, underscoring its potential for clinical translation.

## 3 Discussion

Therapeutic options for p53‐mutant lung cancer remain extremely limited. Conventional chemotherapy and targeted agents often fail to achieve durable efficacy, and strategies capable of restoring p53 function or exploiting its deficiencies are still scarce. In this context, liposomal nanocarriers offer distinct advantages, including improved solubility, stability, and pharmacokinetics, as well as enhanced tumor accumulation via the enhanced permeability and retention (EPR) effect, thereby representing ideal platforms for the codelivery of drugs with complementary mechanisms.

Here, we developed AsAcP@LP, a multifunctional liposomal platform capable of co‐delivering arsenic trioxide (ATO, present as As^5+^) and allicin. ATO has been reported to restore the transcriptional activity of structural mutant p53, thereby reactivating tumor‐suppressive pathways, whereas allicin disrupts redox homeostasis and inhibits ATR to trigger synthetic lethality in DDR‐deficient cells. In vitro, As^5+^ and allicin exhibited pronounced synergistic cytotoxicity in p53‐expressing lung cancer cells at an optimized 7:3 molar ratio, which was successfully recapitulated in the liposomal formulation. In vivo, AsAcP@LP displayed potent antitumor efficacy with favorable stability and tolerability, underscoring its translational potential.

To further elucidate the underlying mechanism, transcriptomic profiling revealed that AsAcP@LP induced a unique gene expression program with minimal overlap with either free drug. The sole common DEG across all comparisons, FAM13B, is linked to protein arginine methylation, a post‐translational modification critical for signal transduction, DDR, transcriptional regulation, and RNA processing [36, 37]. Importantly, AsAcP@LP prominently perturbed genes associated with chromatin remodeling (RSF1, SMARCC2, SMC1A), ubiquitination cascades (USP43, CUL4B, FBXL5), and spliceosome function (SF3B1, PRPF38B, RBM5). RSF1 has been reported to mediate p53‐dependent transcriptional responses to DNA double‐strand breaks by modulating the accessibility of p53/p300 complexes, thereby maintaining genomic stability [38, 39, 40]. These findings raise the possibility that AsAcP@LP may influence p53 signaling and checkpoint activation through chromatin remodeling pathways. In addition, the alterations in ubiquitination‐related genes suggest potential effects on the stability and turnover of DNA repair factors, while perturbation of spliceosome components could contribute to RNA processing defects and R‐loop accumulation, thereby exacerbating replication stress. Disruption of ubiquitination networks and spliceosome components further suggests impaired repair protein stability and RNA processing stress, potentially leading to R‐loop accumulation and replication stress. Collectively, these findings indicate that AsAcP@LP not only induces DNA lesions but also impairs their resolution by restricting chromatin accessibility, destabilizing repair machinery, and aggravating RNA processing defects, thereby amplifying genotoxic stress and providing a molecular basis for the observed synergistic efficacy.

In addition, the mechanistic framework established in this study suggests that the Pro‐ATO/Allicin liposomal platform may have broader applicability beyond the lung cancer models investigated here. Given that TP53 mutations and DNA damage response (DDR) pathway dependencies are prevalent across a wide range of solid tumors—including ovarian, triple‐negative breast, colorectal, and head and neck cancers—this dual‐pathway targeting strategy may be extendable to other malignancies characterized by impaired genome maintenance. In such contexts, the ability of arsenic‐based agents to restore mutant p53 transcriptional activity, combined with allicin‐mediated redox disruption and ATR inhibition, could similarly induce synthetic lethality by overwhelming residual DDR capacity. Nevertheless, tumor heterogeneity, differences in redox buffering capacity, and variability in liposomal delivery efficiency across tissue types may influence therapeutic outcomes. Moreover, the extent to which arsenic‐mediated p53 reactivation occurs in non‐structural p53 mutants or in tumors with coexisting alterations in chromatin regulators remains to be systematically evaluated. These considerations highlight both the translational promise and the limitations of extending this platform to broader oncological settings, underscoring the need for tumor‐type–specific validation and biomarker‐guided patient stratification in future studies.

Taken together, AsAcP@LP integrates mutant p53 reactivation with synthetic lethality targeting DDR vulnerabilities, offering a rational nanotherapeutic strategy for aggressive tumors. Clinically, this approach may be particularly valuable in p53‐mutant cancers that exhibit strong reliance on DDR pathways. Nevertheless, several challenges remain, including the long‐term safety of ATO/allicin codelivery, formulation stability, and potential off‐target effects. Moreover, while our transcriptomic analysis highlights DDR‐related mechanisms, the generalizability of these regulatory nodes across diverse tumor contexts requires further validation. Overall, AsAcP@LP represents a promising nanoplatform for precision oncology, warranting future studies on pharmacodynamics, immunomodulatory effects, and translational evaluation.

## 4 Materials and Methods

### 4.1 Materials

Allicin was purchased from Macklin Biochemical Co., Ltd. (Shanghai, China). Allicin reference standard and octreotide acetate reference standard were obtained from the National Institutes for Food and Drug Control (Beijing, China). Octreotide was supplied by Shanghai Pagon Trading Co. Cholesterol, DSPE‐PEG2000, 1,2‐dioleoyl‐3‐trimethylammonium‐propane (DOTAP), and 1,2‐Dioleoyl‐sn‐glycero‐3‐phosphocholine (DOPC) were purchased from Avanti Biotech Co., Ltd. (Shanghai, China). Sodium arsenite heptahydrate (Na_2_HAsO_4_·7H_2_O) was obtained from Sigma‐Aldrich (USA). Dichloromethane was supplied by Macklin Biochemical Co., Ltd. Methanol was purchased from Thermo Fisher Scientific China (also known as TianDi, USA). Polybrene and puromycin were purchased from Beyotime Biotechnology (Shanghai, China). The P53‐R175H plasmid was synthesized by Beijing Tsingke Biotech Co., Ltd (China). Ultrapure water was used throughout all experiments.

### 4.2 Animal Species and Source

The NCI‐H1299 non‐small cell lung cancer (NSCLC) cell line was obtained from the Cell Resource Center, Shanghai Institutes for Biological Sciences, Chinese Academy of Sciences (RRID:CVCL_0060). The lentiviral particles carrying the P53‐R175H gene was synthesized by Beijing Tsingke Biotech Co., Ltd. The H1299‐P53 cell line, in which the P53‐R175H gene was stably knocked in, was used for comparative studies. Cells were cultured in RPMI‐1640 medium supplemented with 10% fetal bovine serum (FBS) and 1% penicillin–streptomycin at 37°C in a humidified atmosphere containing 5% CO_2_. All animal experiments were approved by the Institutional Animal Care and Use Committee of Zhejiang Chinese Medical University and conducted in accordance with institutional guidelines for the care and use of laboratory animals (approval number: IACUC‐20250908‐16). Male BALB/c nude mice (4–6 weeks old) were purchased from Shanghai SLAC Laboratory Animal Co., Ltd. For tumor xenograft models, H1299 or H1299‐P53 cells were suspended in PBS and subcutaneously injected into the axilla of the mice. Tumor growth was monitored every other day. When tumor volumes reached 50–80 mm^3^, subsequent treatments and analyses were initiated.

### 4.3 Establishment of H1299‐P53 Cells

To generate H1299 cells stably expressing the P53‐R175H mutant, lentiviral transduction was performed. Briefly, adherent H1299 cells were seeded into 24‐well plates at a density of 1 × 10^5^ cells/well approximately 18–24 h prior to infection to ensure a cell density of 2 × 10^5^ cells/well at the time of transduction. On the day of infection, the culture medium was replaced with 2 mL of fresh complete medium containing 6 µg/mL polybrene. Lentiviral particles carrying the P53‐R175H gene were then added to each well, and the cells were incubated at 37°C in 5% CO_2_ for 24 h. After incubation, the virus‐containing medium was removed, and cells were washed twice with PBS, followed by the addition of fresh complete medium for continued culture. Seventy‐two hours post‐infection, puromycin was added to the medium at a final concentration of 2 µg/mL for selection. Selection was continued until all cells in the negative control (non‐transduced) group had died.

### 4.4 Preparation of AsAcP@LP

DOPC, DOTAP, cholesterol, DSPE‐PEG2000, allicin, and octreotide acetate were mixed at various molar ratios and dissolved in a mixture of 10 mL dichloromethane and 5 mL methanol in a 100 mL round‐bottom flask. The mixture was sonicated until fully dissolved, followed by rotary evaporation under reduced pressure at 40°C to remove organic solvents and obtain a uniform lipid film. The dried film was then hydrated with an aqueous PBS solution containing sodium arsenate heptahydrate (Na_2_HAsO_4_·7H_2_O) at room temperature. The resulting liposomal suspension was transferred to an Eppendorf tube and sonicated for 3 min (ultrasonic power: 50%; pulse mode: 3 s on, 7 s off). The suspension was subsequently centrifuged at 8000 rpm for 10 min to remove empty vesicles and phospholipid debris. The final liposomal formulation, co‐loaded with arsenic, allicin, and octreotide, was referred to as AsAcP@LP.

### 4.5 Optimization of AsAcP@LP

To optimize the preparation of AsAcP@LP, the effects of various formulation parameters were systematically investigated, including lipid composition, drug‐to‐lipid ratios, and reaction temperature. Phospholipid‐to‐cholesterol molar ratios (1:1, 2:1, 3:1, 4:1, 5:1), arsenic‐to‐lipid molar ratios (1:1, 1:2, 1:3, 1:4, 1:5), allicin‐to‐lipid mass ratios (1:4, 1:8, 1:12, 1:16, 1:20), and hydration temperatures (20°C, 30°C, 40°C, 50°C, and 60°C) were evaluated with respect to encapsulation efficiency, particle size distribution, and stability. The particle size, polydispersity index (PDI), and zeta potential of the resulting liposomes were measured using a Malvern particle size analyzer (Malvern Instruments, UK). The optimal conditions were identified as a phospholipid‐to‐cholesterol ratio of 3:1, arsenic‐to‐lipid ratio of 1:4, allicin‐to‐lipid ratio of 1:8, and a reaction temperature of 40°C yielding liposomes with uniform particle size (PDI < 0.3), high drug encapsulation efficiency, and good stability.

### 4.6 Drug Loading and Encapsulation Efficiency

To determine the encapsulation efficiency (EE%) and drug loading (DL%) of As in AsAcP@LP, 10 mg of the liposomal formulation was accurately weighed and diluted to 10  mL with ultrapure water in a volumetric flask. The free (unencapsulated) As content was quantified using inductively coupled plasma spectroscopy (ICP). Another 10 mg of AsAcP@LP was weighed and treated with an appropriate amount of nitric acid, followed by sonication and overnight incubation to fully release encapsulated As. The solution was then diluted to 10  mL with ultrapure water, filtered through a 0.45 µm membrane filter, and the total As content was measured by ICP. EE% and DL% were calculated accordingly. For allicin quantification, an equivalent amount of AsAcP@LP was demulsified with methanol. The mixture was filtered through a 0.22  µm polypropylene membrane filter, and the allicin content was determined by high‐performance liquid chromatography (HPLC) to calculate both EE% and DL%.

### 4.7 Characterization of AsAcP@LP

The particle size and zeta potential of AsAcP@LP were measured using a Malvern particle size analyzer. For morphological observation, 10  µL of diluted AsAcP@LP solution was dropped onto a carbon‐coated copper grid and allowed to air‐dry at room temperature. The morphology of the liposomes was then examined using TEM. To further confirm elemental composition and distribution, energy‐dispersive spectroscopy (EDS) was performed on selected nanoparticles observed in the TEM images. The presence and relative distribution of As and S elements were semi‐quantitatively analyzed.

### 4.8 Detection of H_2_S Release from AsAcP@LP

The release of H_2_S from the reaction of allicin within AsAcP@LP and GSH was quantitatively assessed using a colorimetric assay. Briefly, 2 mL of zinc acetate–sodium acetate solution (prepared by dissolving 0.5  g zinc acetate and 0.125  g sodium acetate in 10  mL distilled water) was added to a series of centrifuge tubes. Various volumes (0.00, 0.20, 0.40, 0.60, 0.80, 1.00, 1.50, and 2.00  mL) of the nanoparticle reaction solution (allicin + GSH) were added, and the total volume was adjusted to 6  mL with distilled water. Subsequently, 1  mL of N,N‐dimethyl‐p‐phenylenediamine solution was slowly added to each tube, followed by immediate sealing and gentle inversion. Then, 1  mL of ferric ammonium sulfate solution was added, the tubes were sealed again, thoroughly mixed, and incubated for 30  min at room temperature. Finally, the solutions were diluted to 10  mL with distilled water. The absorbance of the resulting colored product was scanned from 400 to 800  nm using a UV–vis spectrophotometer.

### 4.9 In Vitro As Valence State Transformation

X‐ray photoelectron spectroscopy (XPS) was used to investigate changes in the valence state of As in AsAcP@LP in the presence and absence of GSH. Equal amounts of AsAcP@LP were incubated in PBS or GSH solution for 12 h. After incubation, the supernatants were collected and dropped onto silicon wafers, followed by natural drying at room temperature. The samples were then analyzed by XPS to assess the valence state transformation of arsenic species.

### 4.10 Cellular Uptake

Liposomes were first labeled with the near‐infrared fluorescent probe Cy5.5 (Ex/Em = 680 nm/698 nm). H1299 and H1299‐P53 cells were seeded into confocal culture dishes and incubated overnight. After removing the culture medium, cells were incubated with media containing AsAc@LP‐Cy5.5 and AsAcP@LP‐Cy5.5 for 4 h. Subsequently, cells were fixed with 4% paraformaldehyde in the dark. After staining the nuclei with DAPI (Ex/Em = 340 nm/488 nm) under light‐protected conditions, cellular uptake of different formulations was visualized using a CLSM.

### 4.11 Cytotoxicity Assay

H1299 and H1299‐P53 cells were seeded into 96‐well plates and cultured at 37°C for 24 h. The cells were then incubated with varying concentrations of AsAcP@LP for 24 h at 37°C. Following treatment, MTT solution was added to each well, and the absorbance at 490 nm was measured using a microplate reader. Cell viability of H1299 and H1299‐P53 cells was calculated based on the absorbance values. The same cytotoxicity assay was also performed on normal Beas‐2B and HUVEC cells under identical experimental conditions.

### 4.12 Apoptosis Assay

H1299 and H1299‐P53 cells were seeded in 6‐well plates and cultured for 24 h. The cells were then divided into six groups (control, monotherapy groups (Free As, Free Ac), combination free‐drug group (As + Ac), unmodified liposome group (AsAc@LP), and the optimized liposome formulation group (AsAcP@LP).) and incubated for an additional 24 h. The supernatant was collected and saved in centrifuge tubes. Cells were detached using trypsin, followed by neutralization with culture medium. Cells were centrifuged at 1000 g for 5 min and washed three times with PBS. Apoptosis analysis was performed according to the instructions of the apoptosis detection kit, and subsequently analyzed by flow cytometry.

### 4.13 Live/Dead Cell Staining

H1299 and H1299‐P53 cells were seeded in 6‐well plates and incubated at 37°C overnight. The cells were then divided into six groups: Control, Free As, Free Ac, As + Ac, AsAc@LP, and AsAcP@LP, with each group treated with 20 µg/mL of the corresponding formulation and co‐incubated for 24 h. Afterward, cells were stained with Calcein‐AM (Ex/Em = 494 nm/517 nm) and propidium iodide (PI, Ex/Em = 535 nm/617 nm) for 30 min, followed by observation under a CLSM.

### 4.14 Intracellular H2S Detection

H1299 and H1299‐P53 cells were seeded into 6‐well plates and cultured for 24 h. The cells were then divided into six groups (Control, Free As, Free Ac, As + Ac, AsAc@LP, and AsAcP@LP) and treated for an additional 24 h. After incubation, cells were collected and processed according to the instructions of the H_2_S fluorescent probe kit (Ex/Em = 502 nm/525 nm). Intracellular H_2_S levels were then visualized using a CLSM.

### 4.15 Western Blot Analysis

H1299 and H1299‐P53 cells were seeded in 6‐well plates and treated with different formulations (Control, Free As, Free Ac, As + Ac, AsAc@LP, and AsAcP@LP) for 24 h. After treatment, cells were lysed using RIPA lysis buffer. Total protein concentration was quantified using a BCA protein assay kit. Equal amounts of protein were separated by 15% SDS‐PAGE and transferred onto PVDF membranes. The membranes were blocked with a rapid blocking buffer at room temperature for 1 h and incubated overnight at 4°C with primary antibodies against P53, ATR, phospho‐ATR (P‐ATR), and GAPDH. After washing three times with TBST, membranes were incubated with the corresponding secondary antibodies for 2 h at room temperature. Protein bands were visualized using a chemiluminescence detection system.

### 4.16 Cell Cycle Analysis

Cell cycle distribution was analyzed using a Cell Cycle and Apoptosis Analysis Kit. After treatment, cells were harvested and fixed in 70% ethanol at 4°C overnight. The fixed cells were then stained with PI solution containing RNase A. DNA content was measured by flow cytometry, and the proportions of cells in the G_0_/G_1_, S, and G_2_/M phases were analyzed using FlowJo software.

### 4.17 Comet Assay

DNA damage in cells was evaluated using a comet assay kit, following the manufacturer's instructions. Briefly, collected cells were mixed with low‐melting‐point agarose and spread onto pretreated microscope slides. After lysis, the slides were subjected to DNA unwinding under alkaline conditions, followed by electrophoresis at 25 V for 30 min. The slides were then neutralized, stained with a DNA fluorescent dye provided in the kit, and observed under a fluorescence microscope. Quantitative analysis of DNA damage, including tail length and tail moment, was performed using CASP software.

### 4.18 DNA Damage Assay

DNA damage was assessed using a DNA Damage Detection Kit according to the manufacturer's protocol. Briefly, cells were fixed and permeabilized, followed by incubation with a fluorescently labeled γ‐H2AX antibody. After washing, nuclei were counterstained with DAPI. Fluorescence images were acquired using a CLSM, and the extent of DNA double‐strand breaks was evaluated by quantifying the number of γ‐H2AX foci per nucleus.

### 4.19 Transcriptome Analysis

Total RNA was isolated and purified using TRIzol reagent (Invitrogen, Carlsbad, CA, USA) following the manufacturer's protocol. RNA concentration and purity were determined with NanoDrop ND‐1000 (NanoDrop, Wilmington, DE, USA), and integrity was confirmed using the Agilent 2100 Bioanalyzer (Agilent, CA, USA) with RIN values > 7.0, as well as by electrophoresis on denaturing agarose gels. Poly(A) RNA was enriched from 1 µg of total RNA using Dynabeads Oligo (dT) 25 (Thermo Fisher, CA, USA) through two rounds of selection, and subsequently fragmented into ∼200 nt pieces with the Magnesium RNA Fragmentation Module (NEB, USA) at 94°C for 5–7 min. First‐strand cDNA was synthesized using SuperScript II Reverse Transcriptase (Invitrogen, USA), followed by U‐labeled second‐strand synthesis with E. coli DNA polymerase I (NEB, USA), RNase H (NEB, USA), and dUTP (Thermo Fisher, USA). After end repair and A‐tailing, indexed adapters with T‐overhangs were ligated, and adapter‐ligated fragments were size‐selected with AMPure XP beads. Libraries were treated with heat‐labile UDG (NEB, USA) and amplified by PCR (8 cycles), generating cDNA libraries with an average insert size of 300 ± 50 bp. Paired‐end sequencing (2 × 150 bp) was performed on an Illumina NovaSeq 6000 platform (LC‐Bio Technology, Hangzhou, China).

Raw sequencing reads were quality‐trimmed and aligned to the human reference genome (GRCh38) using HISAT2. Gene expression levels were quantified as fragments per kilobase of transcript per million mapped reads (FPKM) with StringTie, and differential expression analysis was performed using DESeq2. Functional enrichment analyses, including Gene Ontology (GO) and Kyoto Encyclopedia of Genes and Genomes (KEGG) pathway annotation, were conducted to identify relevant biological processes and pathways.

### 4.20 Establishment of Breast Cancer Mouse Model and In Vivo Biodistribution Study

All animal experiments were approved by the Animal Ethics Committee of Zhejiang Chinese Medical University and conducted in accordance with the institutional guidelines for the care and use of laboratory animals. Male BALB/c nude mice (4–6 weeks old) were purchased from Shanghai SLAC Laboratory Animal Co., Ltd. H1299 and H1299‐P53 cell suspensions were subcutaneously injected into the dorsal region of the mice. Tumor growth was monitored every other day, and when tumor volumes reached approximately 50–80 mm^3^, mice were enrolled in subsequent studies. Tumor‐bearing mice were randomly divided into four groups (*n* = 3) and intravenously injected via the tail vein with saline solutions containing either free Cy5.5 or AsAcP@LP‐Cy5.5. At predetermined time points (0.5, 1, 2, 4, 8, 12, and 24 h post‐injection), mice were anesthetized with isoflurane, and the biodistribution of Cy5.5 and AsAcP@LP‐Cy5.5 was monitored using a small animal in vivo imaging system. After the final imaging time point, mice were euthanized by cervical dislocation. Tumors and major organs were harvested and imaged ex vivo to assess the fluorescence intensity of Cy5.5 in individual tissues. Quantitative analysis of fluorescence signals was performed using in vivo imaging software. Additionally, the collected tumors and organs were subjected to further analysis to determine the content and oxidation state of arsenic.

### 4.21 In Vivo Antitumor Efficacy Study

H1299 and H1299‐P53 non‐small cell lung cancer (NSCLC) tumor‐bearing mice were randomly divided into four groups (n = 5 per group): (1) Control group: intravenous injection of saline; (2) Free Ac group: intravenous injection of allicin solution; (3) Free As group: intravenous injection of As^5+^ solution; (4) AsAcP@LP group: intravenous injection of AsAcP@LP formulation. Treatments were administered every other day via tail vein injection at a dose of 4 mg/kg (based on body weight), in a total injection volume of 200 µL. Tumor volumes were measured and recorded daily using caliper measurements and calculated using the standard formula: Volume = (length × width^2^)/2.

In a separate cohort of tumor‐bearing mice, tumors were harvested after 21 days of treatment, fixed in formalin, and processed for histological analysis. Tumor tissues were embedded in paraffin, sectioned, and subjected to hematoxylin and eosin (H&E) staining and terminal deoxynucleotidyl transferase dUTP nick end labeling (TUNEL) assays. The extent of tumor necrosis and apoptosis in different treatment groups was observed under a light microscope.

### 4.22 Biosafety Evaluation

Upon completion of the treatment regimen, blood samples were collected from each group of mice and divided into two portions. One portion was directly analyzed using an automated hematology analyzer to assess hematological parameters. The other portion was centrifuged at 1000 g for 10 min to obtain serum, which was analyzed using an automated biochemical analyzer for liver and kidney function markers, including alanine aminotransferase (ALT), albumin (ALB), and blood urea nitrogen (BUN).

After euthanasia, major organs including the heart, liver, spleen, lungs, and kidneys were harvested from all groups. The tissues were fixed in 4% neutral‐buffered formalin, embedded in paraffin, and sectioned for histological analysis. H&E staining was performed, and histopathological changes, such as inflammation or necrosis, were examined under a light microscope to evaluate potential systemic toxicity.

### 4.23 Statistical Analysis

Statistical Analysis: Prior to statistical analysis, the data were carefully preprocessed to ensure accuracy and reliability. This included removing outliers, imputing missing values, standardizing/normalizing the data (if applicable), and verifying that all data met normality assumptions. Data are presented as mean ± standard deviation (SD). The sample size (n) for each group was determined based on previous studies to ensure sufficient statistical power, with the sample size being consistent across all experimental groups within a given experiment to maintain statistical integrity. For assessing significant differences, one‐way analysis of variance (ANOVA) followed by Tukey's post hoc test was used for normally distributed data.

## Ethics Approval and Consent to Participate

All animal experimental procedures were approved by Animal Ethical and Welfare Committee of Zhejiang Chinese Medical University. (Approval No. IACUC‐20250908‐16).

## Conflicts of Interest

The authors declare no conflicts of interest.

## Supporting information




**Supporting File**: advs73962‐sup‐0001‐SuppMat.docx.

## Data Availability

The data that support the findings of this study are available from the corresponding author upon reasonable request.
