# Correction to “Pro‐ATO/Allicin Liposomes for Dual‐Pathway Targeting of p53‐Mutant Tumors”

**DOI:** 10.1002/advs.75743

**Published:** 2026-05-20

X. Xu, W. Cheng, M. Yang, L. He, W. Ren, J. Chen, L. He, D. Bao, Z. Zhu, L. Wang, Q. Yao, J. G. Piao, “Pro‐ATO/Allicin Liposomes for Dual‐Pathway Targeting of p53‐Mutant Tumors,” *Adv* *anced* *Sci* *ence* (Weinh) 13, no. 18 (2026): e19194, https://doi.org/10.1002/advs.202519194.

**[Description of error]**

There are errors in the numbering and order of author affiliations in the published version of the article, which needs to be corrected.

During the proof stage, we carefully checked the numbering/order of author affiliations and submitted the corresponding revisions. However, it is unfortunate that in the final published version, the affiliation numbering/order was not updated according to the proof corrections and remains inconsistent.

**The numbering and order of the original authors’ affiliations**:

Xiaoling Xu^1,2^, WeiYi Cheng^3^, Menghang Yang^1^, Li He^3^, WeiYe Ren^3^, JingQuan Chen^3^, LiTing He^3^, Dandan Bao^3,4,✉^, Zhihong Zhu^3,✉^, Lixin Wang^5,✉^, Qinghua Yao^6,7,✉^, Ji‐Gang Piao^3,✉^

^1^Department of Radiation Oncology, Shanghai Pulmonary Hospital, Tongji University School of Medicine, Shanghai, China

^2^Zhejiang Provincial Key Laboratory of Diagnosis and Treatment of Thoracic Cancer, Hangzhou, Zhejiang, China

^3^School of Pharmaceutical Sciences, The First Affiliated Hospital of Zhejiang Chinese Medical University, Hangzhou, Zhejiang, China

^4^Department of Dermatology & Cosmetology, The First Affiliated Hospital of Zhejiang Chinese Medical University, Hangzhou, Zhejiang, China

^5^Department of Integrative Medicine, Shanghai Pulmonary Hospital, Tongji University School of Medicine, Shanghai, China

^6^Department of Oncology, The Second Affiliated Hospital of Zhejiang Chinese Medical University, Xinhua Hospital of Zhejiang Province, Hangzhou, Zhejiang, China

^7^Key Laboratory for Research on the Pathogenesis of ‘Inflammation‐Cancer Transformation’ in Intestinal Diseases, Hangzhou, Zhejiang, China

✉Corresponding author.

**The correct numbering and the order of the authors’ affiliations is as follows**:

Xiaoling Xu^3,4^, WeiYi Cheng^1^, Menghang Yang^3^, Li He^1^, WeiYe Ren^1^, JingQuan Chen^1^, LiTing He^1^, Dandan Bao^2,✉^, Zhihong Zhu^1,✉^, Lixin Wang^5,✉^, Qinghua Yao^6,7,✉^, Ji‐Gang Piao^1,✉^

^1^School of Pharmaceutical Sciences, The First Affiliated Hospital of Zhejiang Chinese Medical University, Hangzhou, Zhejiang, China

^2^Department of Dermatology & Cosmetology, The First Affiliated Hospital of Zhejiang Chinese Medical University, Hangzhou, Zhejiang, China

^3^Department of Radiation Oncology, Shanghai Pulmonary Hospital, Tongji University School of Medicine, Shanghai, China

^4^Zhejiang Provincial Key Laboratory of Diagnosis and Treatment of Thoracic Cancer, Hangzhou, Zhejiang, China

^5^Department of Integrative Medicine, Shanghai Pulmonary Hospital, Tongji University School of Medicine, Shanghai, China

^6^Department of Oncology, The Second Affiliated Hospital of Zhejiang Chinese Medical University, Xinhua Hospital of Zhejiang Province, Hangzhou, Zhejiang, China

^7^Key Laboratory for Research on the Pathogenesis of ‘Inflammation‐Cancer Transformation’ in Intestinal Diseases, Hangzhou, Zhejiang, China

✉Corresponding author.

We apologize for this error.

