# Supplementary material for: Pro‐ATO/Allicin Liposomes for Dual‐Pathway Targeting of p53‐Mutant Tumors
Source: Adv Sci (Weinh). 2026 Jan 29;13(18):e19194. doi: 10.1002/advs.202519194 (PMC13042845; doi:10.1002/advs.202519194)
Supplement: Supplementary file 1 — Supporting File: advs73962‐sup‐0001‐SuppMat.docx. [file ADVS-13-e19194-s001.docx]

| **Lec：Chol** | **Size** | **PDI** | **mV** |
| --- | --- | --- | --- |
| 1：1 | 3730.33±639.49 | 0.43 | -7.80 |
| 2：1 | 157.80±3.30 | 0.41 | -9.07 |
| 3：1 | 141.43±2.50 | 0.26 | -0.08 |
| 4：1 | 153.60±2.07 | 0.27 | -0.01 |
| 5：1 | 149.83±0.57 | 0.33 | 0.08 |

**Pro-ATO/Allicin Liposomes for Dual-Pathway Targeting of p53-Mutant Tumors**

Table 1. Optimization of phospholipid-to-cholesterol ratio on particle uniformity and drug encapsulation efficiency.

| **Ac:Lipids** | **Size（nm）** | **PDI** | **mV** |
| --- | --- | --- | --- |
| 1:4 | 242.9±3.3 | 0.48 | -1.14 |
| 1:8 | 165±1.3 | 0.239 | -1.57 |
| 1:12 | 148.4±1.6 | 0.25 | -0.94 |
| 1:16 | 161.6±1.7 | 0.288 | -1.00 |
| 1:20 | 152.57±0.73 | 0.3 | -1.57 |

Table 2. Optimization of allicin-to-lipid ratio on particle uniformity and drug encapsulation efficiency.

| **As:Lipids** | **Size（nm）** | **PDI** | **mV** |
| --- | --- | --- | --- |
| 1:1 | 242.9±3.3 | 0.48 | -7.46 |
| 1:2 | 165±1.3 | 0.2396 | -8.59 |
| 1:3 | 148.4±1.6 | 0.25 | -7.43 |
| 1:4 | 161.6±1.7 | 0.288 | -8.91 |
| 1:5 | 152.57±0.73 | 0.3 | -10.02 |

Table 3. Optimization of arsenic-to-lipid ratio on particle uniformity and drug encapsulation efficiency.

| **Temperature** | **Size** | **PDI** | **mV** |
| --- | --- | --- | --- |
| 20 ℃ | 192.27±2.35 | 0.43 | -1.70 |
| 30 ℃ | 140.53±0.85 | 0.33 | -1.41 |
| 40 ℃ | 138.70±1.61 | 0.30 | -1.98 |
| 50 ℃ | 129.53±1.15 | 0.29 | -2.21 |
| 60 ℃ | 127.80±0.40 | 0.28 | -0.95 |

Table 4. Optimization of temperature on particle uniformity and drug encapsulation efficiency.


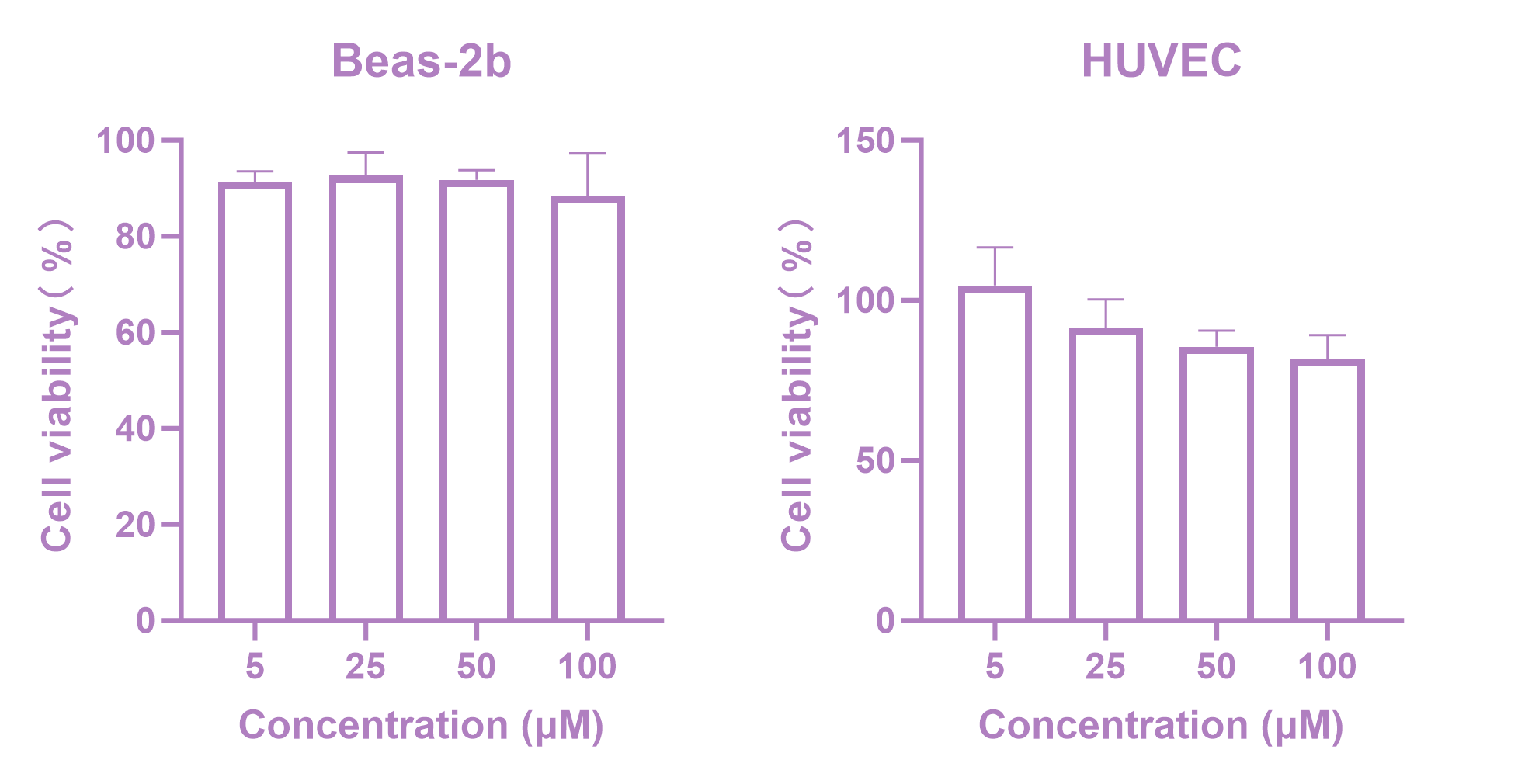


**Fig. S1** Cell viability of Beas-2B and HUVEC cells following 24 h incubation with different concentrations of AsAcP@LP. (n = 3).


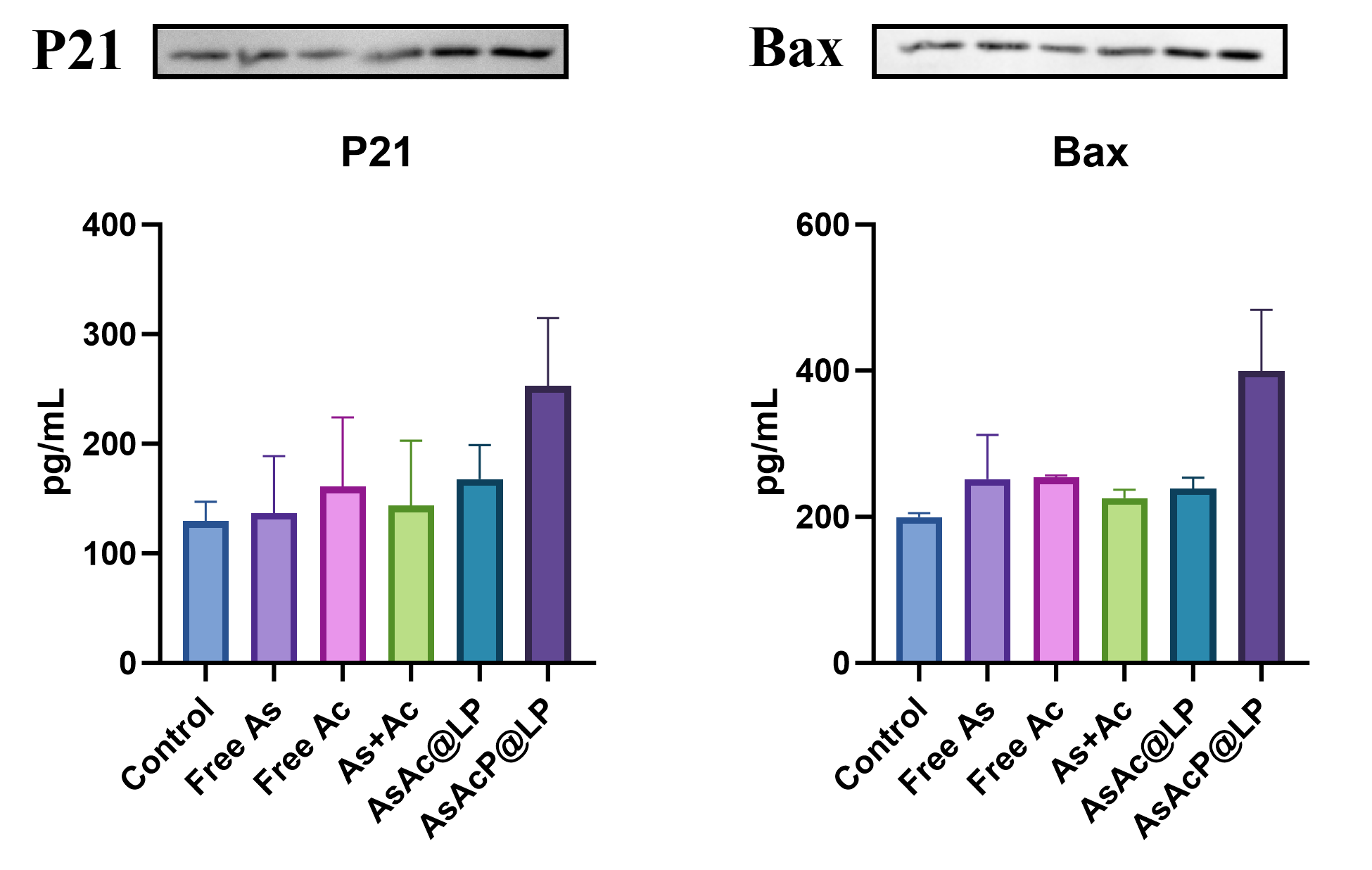


**Fig. S2** Quantification of p21 and Bax protein expression in H1299-P53 cells following treatment with AsAcP@LP.


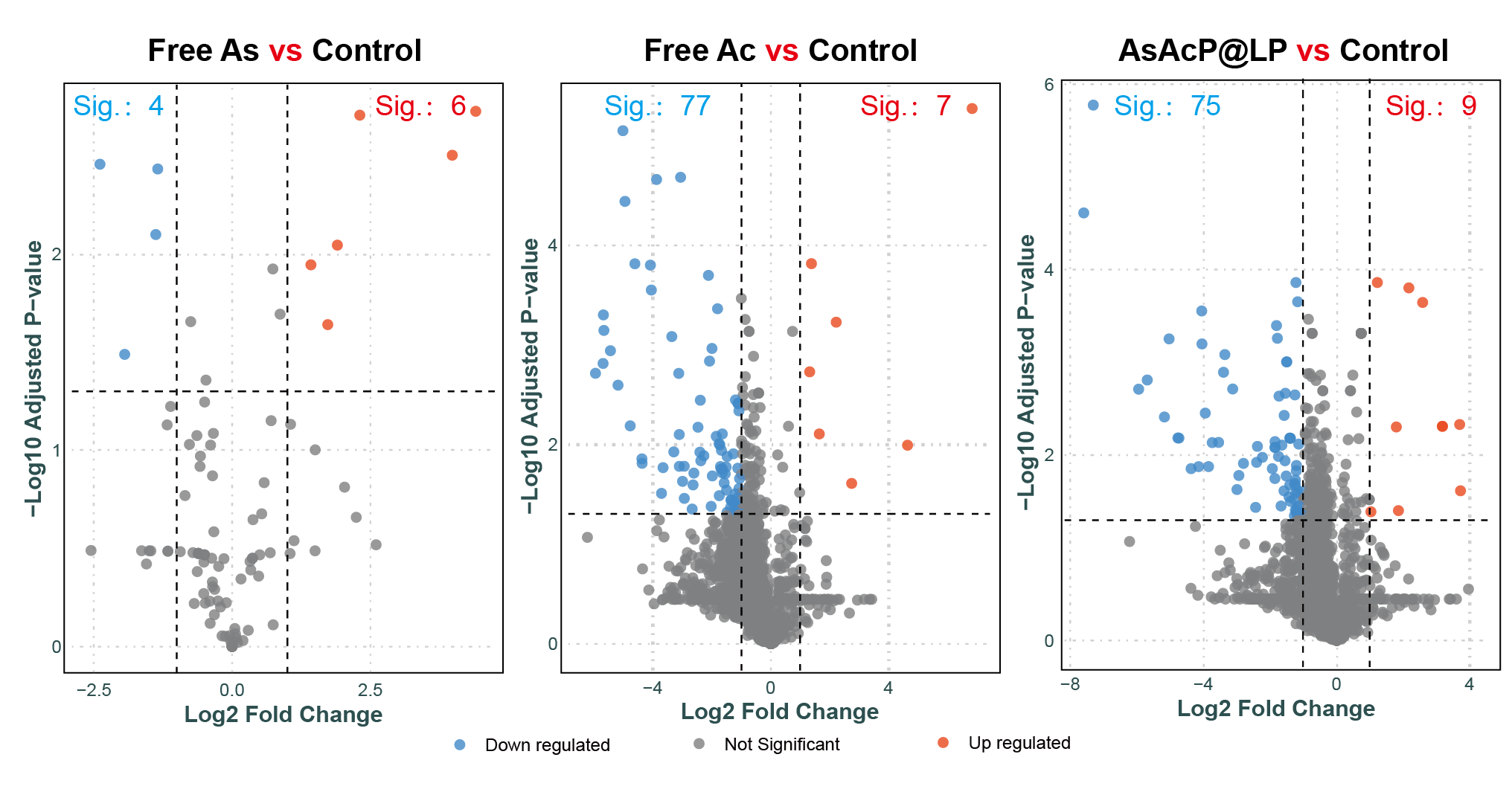


**Fig. S3** Volcano plots depicting differentially expressed genes (DEGs) between treatment and control groups, highlighting the distinct transcriptional responses induced by each formulation.


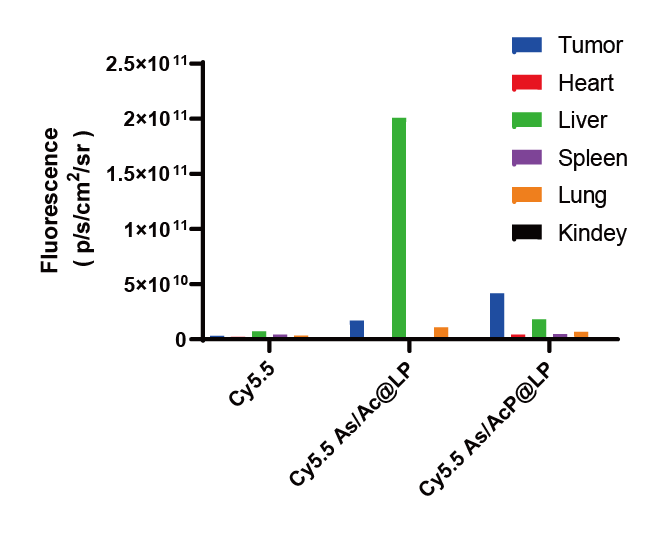


**Fig. S4** Fluorescence intensity of excised organs from different groups, showing biodistribution patterns and tumor-targeting efficiency of liposomal formulations.


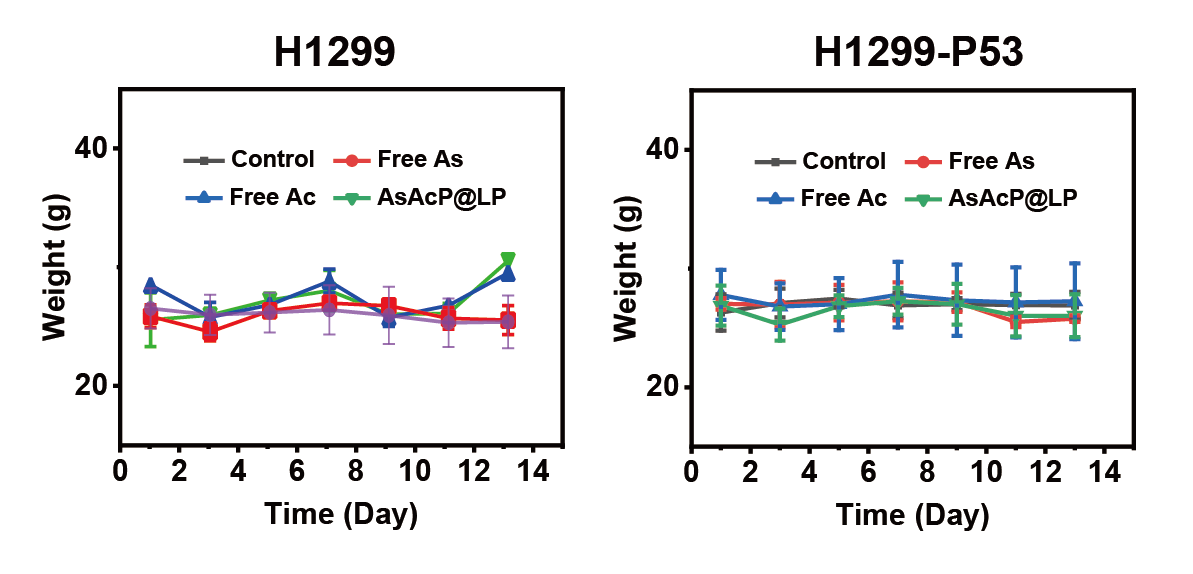


**Fig. S5** Changes in body weight of mice across different treatment groups during the dosing period, indicating overall systemic tolerance and absence of significant treatment-related toxicity.


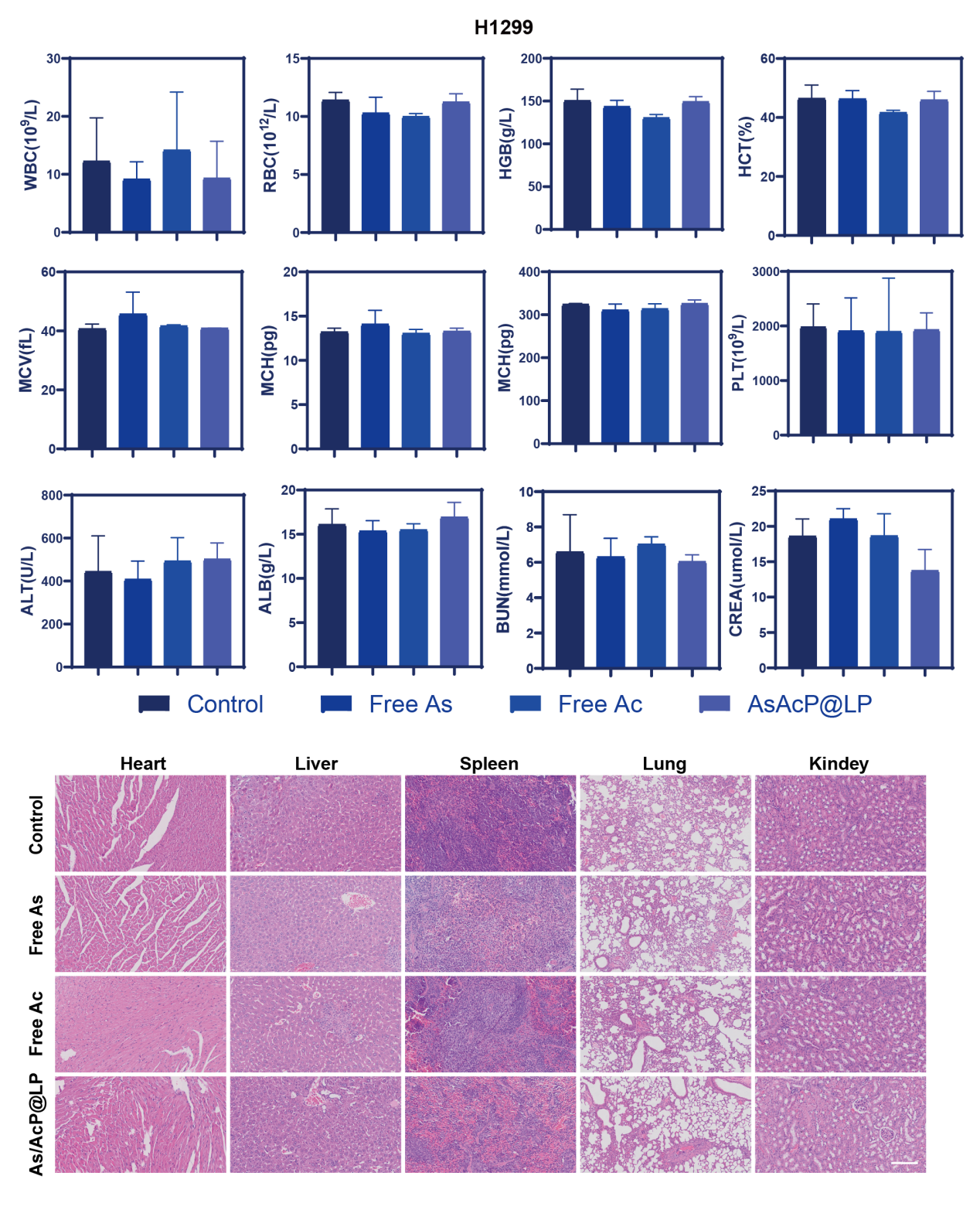


**Fig. S6** Comprehensive biosafety assessment including hematological parameters, serum biochemistry, and histopathological examination of major organs, demonstrating favorable safety profile of AsAcP@LP compared with controls.


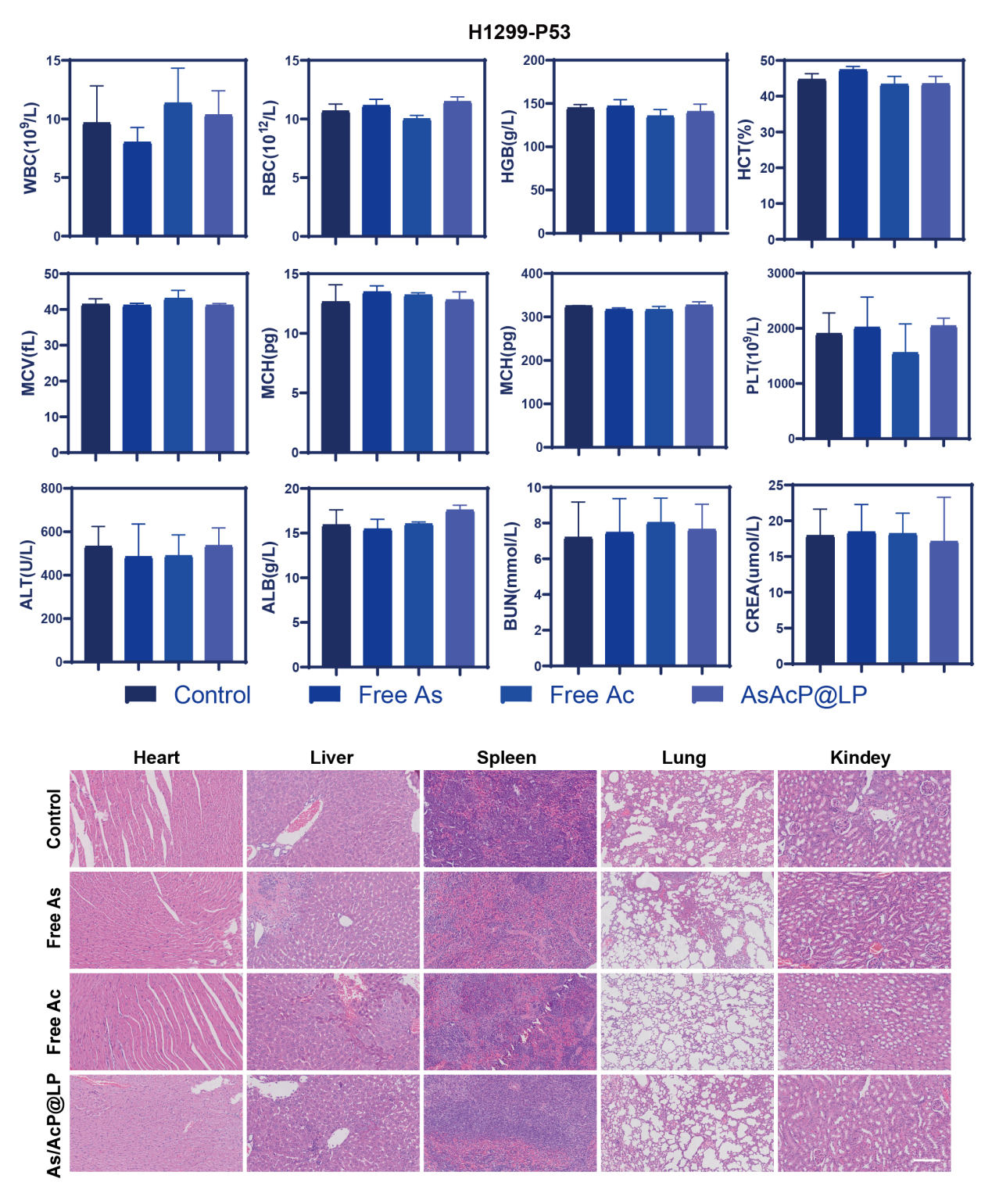


**Fig. S7** Comprehensive biosafety assessment including hematological parameters, serum biochemistry, and histopathological examination of major organs, demonstrating favorable safety profile of AsAcP@LP compared with controls.
